# Shaping and Patterning Supramolecular Materials—Stem Cell-Compatible Dual-Network Hybrid Gels Loaded with Silver Nanoparticles

**DOI:** 10.1021/acsbiomaterials.1c01560

**Published:** 2022-04-02

**Authors:** Carmen C. Piras, Clare S. Mahon, Paul G. Genever, David K. Smith

**Affiliations:** † Department of Chemistry, University of York, Heslington, York YO10 5DD, United Kingdom; ‡ Department of Biology, University of York, Heslington, York YO10 5DD, United Kingdom

**Keywords:** antibacterial, gel, nanoparticles, self-assembly, silver, stem cells

## Abstract

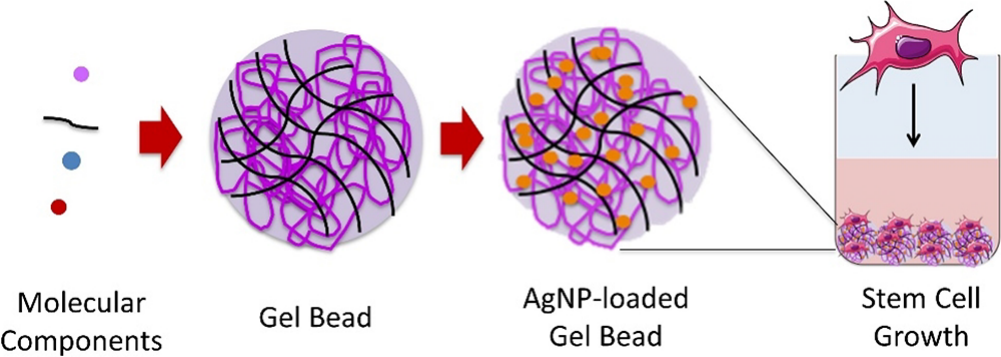

Hydrogels with spatio-temporally controlled properties are appealing materials for biological and pharmaceutical applications. We make use of mild acidification protocols to fabricate hybrid gels using calcium alginate in the presence of a preformed thermally triggered gel based on a low-molecular-weight gelator (LMWG) 1,3:2:4-di(4-acylhydrazide)-benzylidene sorbitol (DBS-CONHNH_2_). Nonwater-soluble calcium carbonate slowly releases calcium ions over time when exposed to an acidic pH, triggering the assembly of the calcium alginate gel network. We combined the gelators in different ways: (i) the LMWG was used as a template to spatially control slow calcium alginate gelation within preformed gel beads, using glucono-δ-lactone (GdL) to lower the pH; (ii) the LMWG was used as a template to spatially control slow calcium alginate gelation within preformed gel trays, using diphenyliodonium nitrate (DPIN) as a photoacid to lower the pH, and spatial resolution was achieved by masking. The dual-network hybrid gels display highly tunable properties, and the beads are compatible with stem cell growth. Furthermore, they preserve the LMWG function of inducing in situ silver nanoparticle (AgNP) formation, which provides the gels with antibacterial activity. These gels have potential for eventual regenerative medicine applications in (e.g.) bone tissue engineering.

## Introduction

Low-molecular-weight gelators (LMWGs) are small molecules that self-assemble in water through noncovalent interactions in response to gelation triggers (e.g., heat, pH, and light), yielding supramolecular hydrogels.^1−3^ Research on LMWGs has seen a dramatic expansion in the past decade, rapidly moving from the discovery of new gelators to investigation of their applications in (e.g.) tissue engineering,^4−7^ drug delivery,^8−11^ and sensors and electronics.^4,12^ Despite presenting promising opportunities, however, their use remains very limited compared to polymer gelators (PGs).^13−15^ This is, at least in part, due to the poor mechanical properties of LMWG hydrogels, which can make it difficult to generate robust materials or impose desired shapes and patterns.^16,17^ Combining LMWGs with PGs is one strategy to increase the structural and functional complexity of hydrogel materials.^18,19^ Synergistic interactions between the two components provide new opportunities in terms of gel stability and mechanical properties, expanding the pool of potential applications. Using orthogonal gelation mechanisms allows the development of multifunctional hybrid gels with programmable self-assembly.

**Scheme 1:**
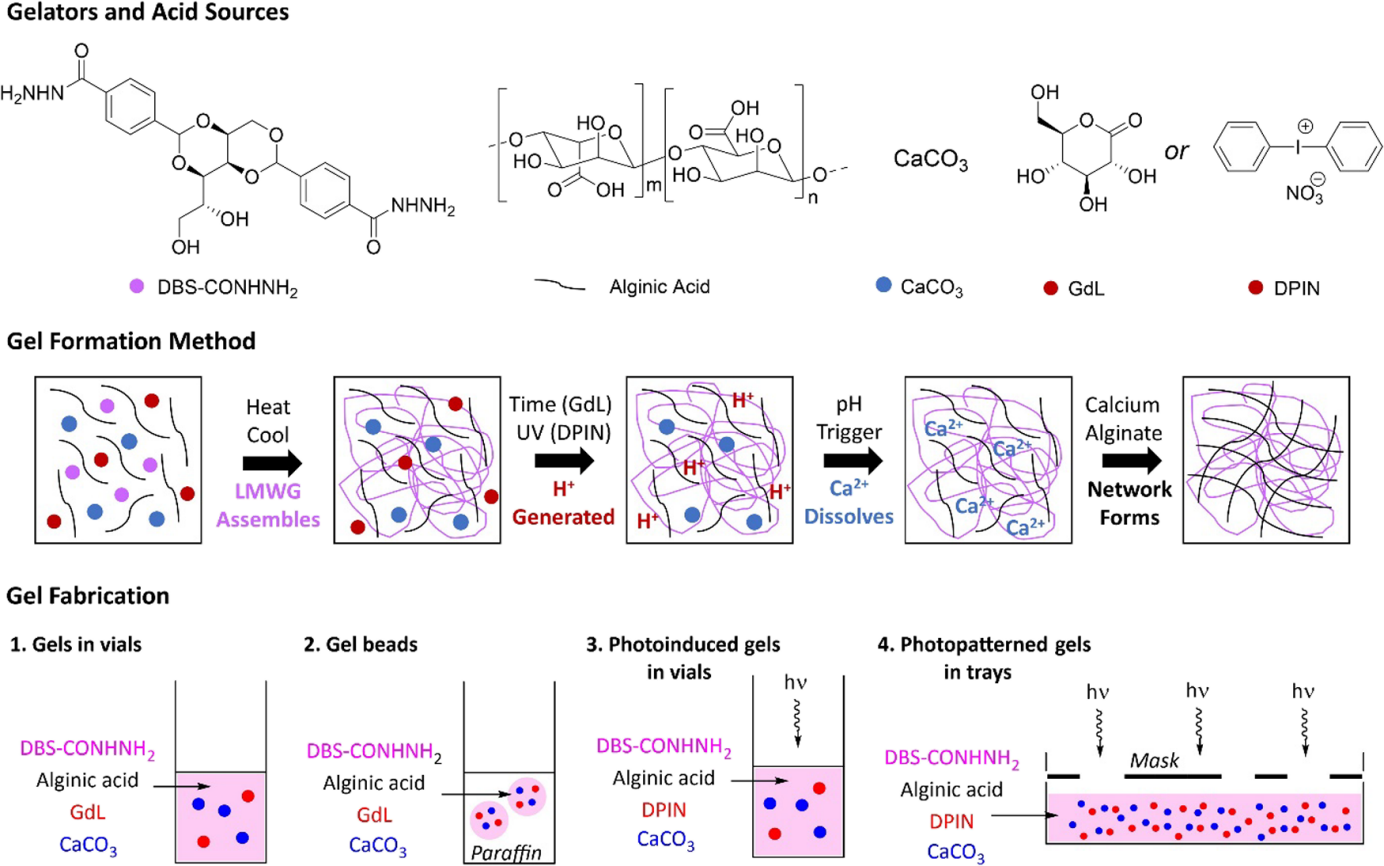
The Gelators and Acid Sources, Gel Formation Method, and Gel Fabrication (Top) Chemical structures of DBS-CONHNH_2_, alginic acid, calcium carbonate, glucono-δ-lactone (GdL), and diphenyliodonium nitrate (DPIN). (Center) Schematic representation of DBS-CONHNH_2_/calcium alginate dual-network hybrid gel formation by cross-linking with CaCO_3_ and either GdL or DPIN. The LMWG is initially assembled via a thermally triggered process, and then, the slow proton release from GdL (over time) or DPIN (triggered by UV light) lowers the pH and causes Ca^2+^ ions to dissolve and subsequently cross-link the alginic acid polymer chains. (Bottom) Approaches to gel fabrication reported in this paper: (1) gelation of alginate in vials of a preformed DBS-CONHNH_2_ gel using GdL activation of CaCO_3_, (2) gelation of alginate in preformed DBS-CONHNH_2_ gel beads using GdL activation of CaCO_3_, (3) gelation of alginate in vials of a preformed DBS-CONHNH_2_ gel using photoinduced DPIN activation of CaCO_3_, and (4) gelation of alginate in trays of a preformed DBS-CONHNH_2_ gel using photoinduced DPIN activation of CaCO_3_

In this regard, we recently explored multicomponent hybrid gels based on the LMWG 1,3:2:4-di(4-acylhydrazide)-benzylidene sorbitol (DBS-CONHNH_2_; Scheme 1) and the polysaccharide calcium alginate (Scheme 1).^20−23^ DBS-CONHNH_2_ is a thermally triggered LMWG that self-assembles in response to heat–cool cycles, giving biocompatible hydrogels that have been employed in a variety of ways including drug delivery, cell culture, and environmental remediation.^24−29^ The biopolymer alginate forms hydrogels when cross-linked with bivalent cations (e.g., Ca^2+^ from CaCl_2_).^30−34^ Combining the two gelators allowed us to impose a spherical shape on the LMWG while keeping its functionality, leading to a rare example of LMWG hydrogel beads.^20−23,35−39^

**Figure 1:**
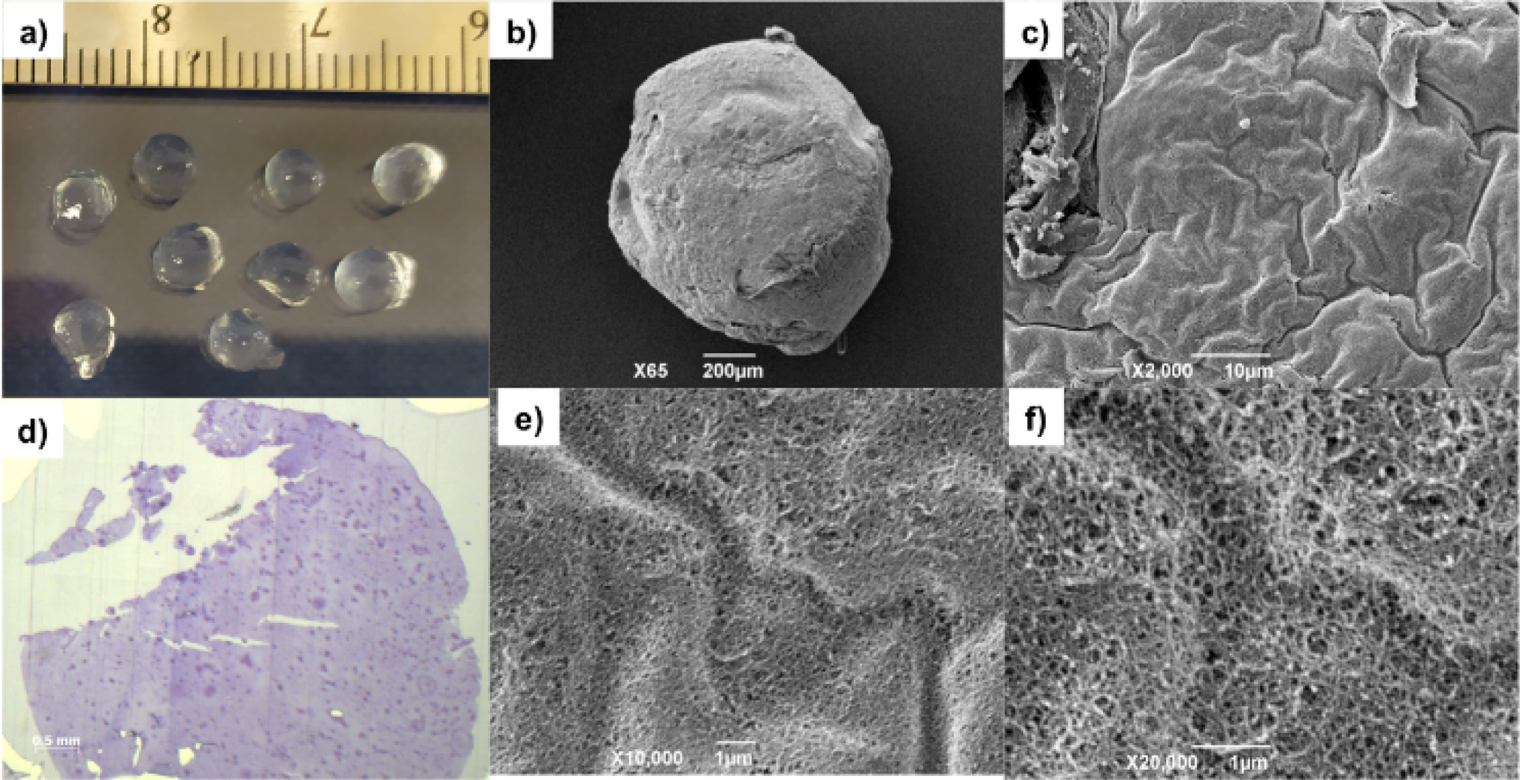
(a) Photographic image of DBS-CONHNH_2_/alginate gel beads. (b,c) SEM images of the DBS-CONHNH_2_/alginate gel bead and the gel bead surface, scale bars of 200 and 10 μm, respectively. (d) Optical microscopy image of the DBS-CONHNH_2_/alginate gel bead cross section embedded in resin and stained with toluidine blue, scale bar of 0.5 mm. (e,f) SEM images of the DBS-CONHNH_2_/alginate gel bead cross section, scale bars of 1 μm.

It is known that calcium alginate assembly can be controlled using different calcium sources.^30−34^ The most common cross-linker for alginate is CaCl_2_. Its water solubility means that when sodium alginate is combined with an aqueous solution of CaCl_2_, the Ca^2+^ ions are immediately available to form ionic interchain bridges between the polymer chains. Since gelation happens very quickly, it can yield inhomogeneous gels. By contrast, nonwater-soluble calcium salts (e.g., CaCO_3_ and CaSO_4_) can slowly release calcium ions over time when exposed to an acidic pH, resulting in more homogeneous gels, and this approach has been of considerable use in the development of alginate PGs.^40−48^

Given our interest in imposing well-defined shapes and structures on LMWGs, we hypothesized that combining our LMWG DBS-CONHNH_2_ with pH-triggered assembly of calcium alginate would give new methods for controlling the fabrication of dual-network hybrid gels. This paper explores the assembly of DBS-CONHNH_2_/alginate gels by pH-triggered release of Ca^2+^ ions from CaCO_3_ achieving both spatial and temporal control over the resulting materials. In particular, we reasoned that the pH control of PG assembly would allow us to photopattern our hybrid gels into multidomain materials within trays—something that cannot be achieved when using CaCl_2_ to trigger alginate assembly. Furthermore, we wanted to demonstrate that the LMWG would retain its unique properties within these shaped materials, in particular the ability to reduce precious metals in situ.^21,25^ We hypothesized that such materials should be compatible with human mesenchymal stem cells as a result of the benign LMWG/PG combination and that the presence of AgNPs may endow such gels with antibacterial properties.

## Results and Discussion

### Simple DBS-CONHNH_2_/Alginate CaCO_3_ Gels in Sample Vials

#### Preparation of Gels in Vials

Initially, we synthesized the two-component hybrid gels in sample vials to gain a basic understanding of the use of glucono-δ-lactone (GdL) as an acid source, along with CaCO_3_ to release calcium ions and hence trigger the cross-linking of calcium alginate in these materials. Glucono-δ-lactone is a cyclic ester that slowly hydrolyzes in water and induces gradual pH lowering, and it has been used before to achieve the release of Ca^2+^ from solid CaCO_3_ and generate homogeneous calcium alginate gels in situ.^40^ We therefore combined DBS-CONHNH_2_ (0.3% wt/vol, 6.3 mM), CaCO_3_ (0.15% wt/vol, 15 mM), and GdL (0.8% wt/vol, 45 mM) with an aqueous solution of sodium alginate (0.5% wt/vol), heated until dissolution of the LMWG (insoluble CaCO_3_ remained) and then cooled. Within 20 min, an initial gel formed, which was attributed to the thermally induced assembly of the DBS-CONHNH_2_ network. The gel was then left undisturbed overnight in which GdL hydrolysis and pH lowering (Figures S1 and S2) gave rise to the slow release of Ca^2+^ ions, which were then able to cross-link the alginate PG (Scheme 1). The final pH of the gel was 6–7.

#### Characterization of Gels in Vials

Macroscopically, the gel–sol transition temperature of the DBS-CONHNH_2_ gel (0.4% wt/vol, *T*_gel_ of 86 °C) measured by the tube inversion method increased to higher temperatures (96 to >100 °C) in the presence of increasing alginate loadings (0.1 to 1.0% wt/vol; Table S1). This was also previously observed for the DBS-CONHNH_2_/alginate hybrid gels prepared using CaCl_2_ as a cross-linker,^20^ thus confirming that the PG is indeed forming within the dual-network hybrid gel and improving the thermal stability.

The mechanical properties of the DBS-CONHNH_2_/alginate hybrid gels were evaluated by oscillatory rheology with a parallel plate geometry in triplicate. As expected, the elastic modulus of the DBS-CONHNH_2_ gel (0.4% wt/vol, G′ = 800 Pa, Figure S9) progressively increases (to 3360, 3870, 4090, and 4430 Pa) in the presence of increasing alginate loadings (0.1, 0.3, 0.5, and 1.0% wt/vol) in the hybrid gels (0.8% wt/vol GdL and 0.15% wt/vol CaCO_3_; Table S2 and Figures S15–S18). These values are also significantly higher than the *G*′ values of the gels formed under the same conditions by calcium alginate alone (299, 424, and 463 Pa; Table S2 and Figures S10–S14), demonstrating the greater stiffness of the dual-network hybrid materials. Compared to the DBS-CONHNH_2_/alginate gels cross-linked with CaCl_2_ that we previously reported,^20^ the gels cross-linked with CaCO_3_ have lower *G*′ values.

To check the effect of CaCO_3_ concentration on the mechanical properties of the gels, we compared the elastic moduli of the hybrid gels prepared at varying CaCO_3_ concentrations (0.05, 0.15, and 0.3% wt/vol; 5, 15, and 30 mM, respectively) and using equal amounts of the two gelators (0.3% wt/vol) and a fixed GdL concentration (0.8% wt/vol, 45 mM). The *G*′ of the gels prepared at the lowest cross-linker concentration (0.05% wt/vol) was 2110 Pa, which increased significantly to 3870 Pa when the gels were prepared with a CaCO_3_ concentration of 0.15% wt/vol (Table S2 and Figure S19). A further increase in CaCO_3_ concentration (0.3% wt/vol) did not have any significant effect on the gel elastic modulus (*G*′ = 3340 Pa; Table S2 and Figure S20). Although this value appears slightly lower than the *G*′ observed using 0.15% wt/vol CaCO_3_, the errors in *G*′ determination mean that this is not a significant difference. The lack of an increase in *G*′ on further increasing CaCO_3_ loading can be explained considering that, at a GdL loading of 0.8% wt/vol (45 mM), only some of the CaCO_3_ (0.3% wt/vol, 30 mM) can be converted into Ca^2+^ and H_2_CO_3_. Specifically, given that 2 equiv of H^+^ are required to react with CaCO_3_, 45 mM GdL can only fully react with 22.5 mM CaCO_3_ (not 30 mM). Increasing the CaCO_3_ concentration further therefore does not increase the elastic modulus of the gels because the H^+^ concentration is not able to release more Ca^2+^.

To explore whether a higher GdL concentration could further improve gel stiffness, we studied the gels using a fixed CaCO_3_ concentration (0.15% wt/vol, 15 mM) and different amounts of GdL (0.8, 1.0, and 1.2% wt/vol; 45, 56, and 67 mM, respectively). The gels prepared with 1.0% wt/vol GdL showed a significantly higher *G*′ (6190 Pa; Table S2 and Figure S21) compared to the gels prepared using 0.8% wt/vol (3870 Pa; Table S2 and Figure S17), suggesting some benefit to a greater excess of GdL. However, a further increase to 1.2% wt/vol of GdL did not significantly improve the gel stiffness (*G*′ = 6280 Pa; Table S2 and Figure S22), likely because at these higher GdL loadings, all of the CaCO_3_ has already been converted into Ca^2+^ and H_2_CO_3_. As expected therefore, for optimum gel performance, the concentrations of GdL and CaCO_3_ must be controlled such that the former can fully activate the latter. These studies demonstrate the tunable mechanical properties of the hybrid gels in response to different parameters. Modifications of these factors allow the design of versatile soft materials with desired stiffness for specific applications.

The supramolecular interactions between DBS-CONHNH_2_ and alginate were studied using IR spectroscopy on the xerogels prepared in sample vials using different alginate loadings and HCl (1 M, 15 μL) instead of GdL to lower the pH. In the presence of alginate, the O–H (3278 cm^–1^) and the N–H (3186 cm^–1^) stretching bands of DBS-CONHNH_2_ were broadened, whereas the C=O band of alginate (1590 cm^–1^) shifts to higher wavenumbers in the presence of the LMWG, suggesting noncovalent interactions between the two gel networks (Figure S5).

Transmission and scanning electron microscopy (TEM and SEM) performed on the gels (Figures S6–S8) indicated the assembly of nanofibrillar networks. This method visualizes the fibers formed by both LMWG and PG networks, and although it cannot fully differentiate between them, the DBS-CONHNH_2_ gel alone comprised slightly narrower fibers (the most common fiber diameter was 10–20 nm), while once calcium alginate was also present in the hybrid gel, the observed fibers were slightly wider (the most common fibers were 21–30 nm), an effect more marked in the calcium alginate-only gel. This indicates that the DBS-CONHNH_2_ nanofibers formed via the heat–cool cycle are narrower than the GdL-induced calcium alginate nanofibers.

In summary, as expected, gels with interpenetrated gel networks could be made in vials using this fabrication technique, with the presence of calcium alginate acting to thermally stabilize the gels and provide a rheological stiffening effect. We therefore went on to explore the extent to which this approach could be used to create hybrid gels with predefined shapes and patterns.

### Shaped DBS-CONHNH_2_/Alginate CaCO_3_ Gel Beads

#### Preparation of Gel Beads

We explored the fabrication of DBS-CONHNH_2_/alginate CaCO_3_ gel beads using the LMWG as a template to impose a spherical shape. The hybrid beads were prepared through a one-step emulsion method by combining DBS-CONHNH_2_ (0.3% wt/vol, 6.3 mM), CaCO_3_ (0.15% wt/vol, 15 mM), and glucono-δ-lactone (GdL, 0.8% wt/vol, 45 mM) with an aqueous solution of sodium alginate (0.5% wt/vol). The resulting suspension was heated until complete dissolution of the LMWG (insoluble CaCO_3_ remained) and then added dropwise (20 μL drops) to a paraffin oil bath. As the system cooled as droplets suspended in paraffin, the DBS-CONHNH_2_ network rapidly assembled. The droplets were left undisturbed overnight to allow cross-linking of the alginate chains on GdL hydrolysis, with pH lowering releasing Ca^2+^ ions (Scheme 1). After 24 h, the gel beads were collected, and the residual paraffin oil was removed through multiple washings with petroleum ether, ethanol, and water. The gel beads have a diameter of 3.0–3.5 mm (Figure 1a), which could be modified by adding different volumes of the gelator mixture to the paraffin oil.

The two gelators play cooperative roles in this fabrication method:

(i) The LMWG acts as a mold to direct alginate gelation within the preformed, thermally induced LMWG spheres; indeed, in the absence of the LMWG, under the same conditions, the alginate droplets coalesce in paraffin oil before self-assembly, leading to unshaped gels.
(ii) The calcium alginate cross-linking acts to stabilize the DBS-CONHNH_2_ gel beads, which, otherwise, would be too fragile to be handled and preserved over time.

#### Characterization of Gel Beads

Since calcium alginate gelation should be homogeneous within the preformed DBS-CONHNH_2_ gel bead template, induced as Ca^2+^ is produced by slow acidification, we expected that interpenetrating gel networks would be formed through the volume of the gel beads. This was confirmed by optical microscopy of a cross section of a gel bead embedded in resin and stained with toluidine blue (Figure 1d), which showed a broadly uniform texture. There were some darker blue marks through the image—we suggest that they result from undissolved CaCO_3_ or indicate the points from which CaCO_3_ was dissolved—they were not previously observed when CaCl_2_ was used to create gel beads.^20^ To obtain insight into the nanofibrillar network within the gel beads, we performed scanning electron microscopy (SEM). The imaging indicated a wrinkled, densely packed surface (Figure 1b,c) and an extended nanofibrillar network in the cross section (Figure 1e,f), consistent with the homogeneous incorporation of self-assembled gelators.

To verify that self-assembly had taken place for both gelators, we transferred 5 gel beads into an NMR tube in D_2_O, with DMSO as an internal standard. If an LMWG is self-assembled into solid-like nanofibers, then its signals cannot be observed by ^1^H NMR spectroscopy,^49−51^ whereas if it remains in the mobile liquid-like phase, then it will exhibit NMR resonances. For the LMWG, the lack of the characteristic DBS-CONHNH_2_ peaks in the gel beads confirmed its self-assembled nature (Figure S3). The alginate ^1^H NMR signals overlap with those of GdL; therefore, we could not make quantitative conclusions for the PG.

To calculate the exact amount of DBS-CONHNH_2_ incorporated into each gel bead and demonstrate the efficiency of the fabrication method, we performed another simple ^1^H NMR experiment. Ten gel beads were dried under vacuum, and the resulting solid was dissolved in DMSO-*d*_6_, which dissolves the LMWG but not alginate, and CH_3_CN (1.4 μL) was added as an internal standard. The sample was analyzed by ^1^H NMR spectroscopy, and the amount of DBS-CONHNH_2_ incorporated into each gel bead was calculated by comparison of the integrals of the LMWG aromatic signals (δ = 7.53 and 7.83 ppm) with the CH_3_CN methyl group (δ = 2.09 ppm; Figure S4). Considering that ca. 1.3 μmol was used to prepare 10 gel beads, if all DBS-CONHNH_2_ was incorporated, then each bead should contain c.a. 0.13 μmol of the LMWG. This experiment showed that 0.13 μmol was indeed encapsulated into each gel bead, confirming the efficiency of this fabrication method.

In summary, therefore, this pH-controlled approach is an effective way of fabricating hybrid LMWG/PG gel beads with well-defined shapes and interpenetrated LMWG/PG networks.

### Photoinduced DBS-CONHNH_2_/Alginate CaCO_3_ Gels

#### Preparation of Photoinduced Gels in Vials

We next decided to explore the UV-triggered pH activation of CaCO_3_ to demonstrate that hybrid gels can also be fabricated in vials by combining orthogonal thermal and UV triggers. A photoacid generator such as diphenyliodonium nitrate (DPIN, Scheme 1) is an effective way of lowering the pH on photoirradiation, with the potential to release Ca^2+^ and trigger calcium alginate assembly.^52−56^ Using photoirradiation opens the possibility of spatial resolution, giving rise to patterned materials; photopatterned hybrid materials containing LMWGs have only rarely been reported,^57−64^ making this approach of considerable interest.

We first explored the applicability of our method to cross-link the PG alone in order to gain some benchmark characterization data. Photoactivated gels in vials were obtained by combining sodium alginate (0.4–1.3% wt/vol) with CaCO_3_ (0.15% wt/vol, 15 mM) and the photoacid generator diphenyliodonium nitrate (DPIN, 0.8% wt/vol, 23.3 mM) followed by exposure to UV light under a high-intensity UV lamp. After 2 h, self-supporting gels were obtained (Figure S23), thus confirming that Ca^2+^ release and polymer cross-linking could potentially be triggered by photoirradiation.

We therefore decided to apply this procedure to create DBS-CONHNH_2_/alginate hybrid gels in vials, which were fabricated as follows. DBS-CONHNH_2_ (0.3% wt/vol, 6.3 mM) was dispersed in water and combined with CaCO_3_ (0.15% wt/vol, 15 mM), DPIN (0.8% wt/vol, 23.3 mM), and sodium alginate (0.5% wt/vol). The mixture was heated until complete dissolution of the LMWG (insoluble CaCO_3_ remained), allowed to cool for 15–20 min, and then exposed to UV light for 2 h to give self-supporting UV-activated gels (Figure S23).

#### Characterization of Photoinduced Gels in Vials

To confirm that the two gelators were in a self-assembled state at the end of the experiment, we performed a simple NMR experiment. The DBS-CONHNH_2_/alginate gel was prepared as described above in an NMR tube using D_2_O instead of water. After exposure to UV light, a ^1^H NMR spectrum was recorded, which showed no signal for either the LMWG or the PG, thus confirming that both the components could self-assemble into gel networks under these conditions (Figure S24). Pleasingly, this confirmed that lowering the pH in this way indeed led to Ca^2+^ release and alginate cross-linking.

The thermal properties of the gels were consistent with calcium alginate formation having taken place (Table S3). The mechanical properties of the photoactivated gels were then studied by oscillatory rheology and compared to those of the gels prepared using GdL as a pH activator. The photoactivated calcium alginate gel (0.6% wt/vol) has an elastic modulus of only 32.5 Pa (Table S4 and Figure S29), which is much lower than the *G*′ of the corresponding gel prepared using GdL (*G*′ = 424 Pa). The hybrid gel prepared by photoactivation using an equal amount of the two gelators (0.3% wt/vol) also showed a much lower *G*′ value (117 Pa, Table S4 and Figure S28) than the gel prepared with GdL (3870 Pa). The photoactivated gels using DPIN as a proton source are therefore much less stiff, and much softer, than those formed by GdL activation. We previously observed similar behavior for other DPIN photoactivated gels,^57^ and we attributed this to (i) less effective acidification and (ii) the formation of iodobenzene as a byproduct, which could weaken the gel. Interestingly, the photoactivated gels were also more elastic and less brittle than the gels prepared with GdL (Table S3 and Figures S28 and S29). The cross-over point (*G*′ = *G*″) for the photoactivated alginate gels was relatively high, at 79.3% shear strain, whereas it was only around 4.0% for the gels prepared with GdL. The DBS-CONHNH_2_/alginate gel prepared using DPIN had a linear viscoelastic region (LVER) that ends at ca. 25%, whereas it ends at ca. 10% when prepared using GdL.

**Figure 2:**
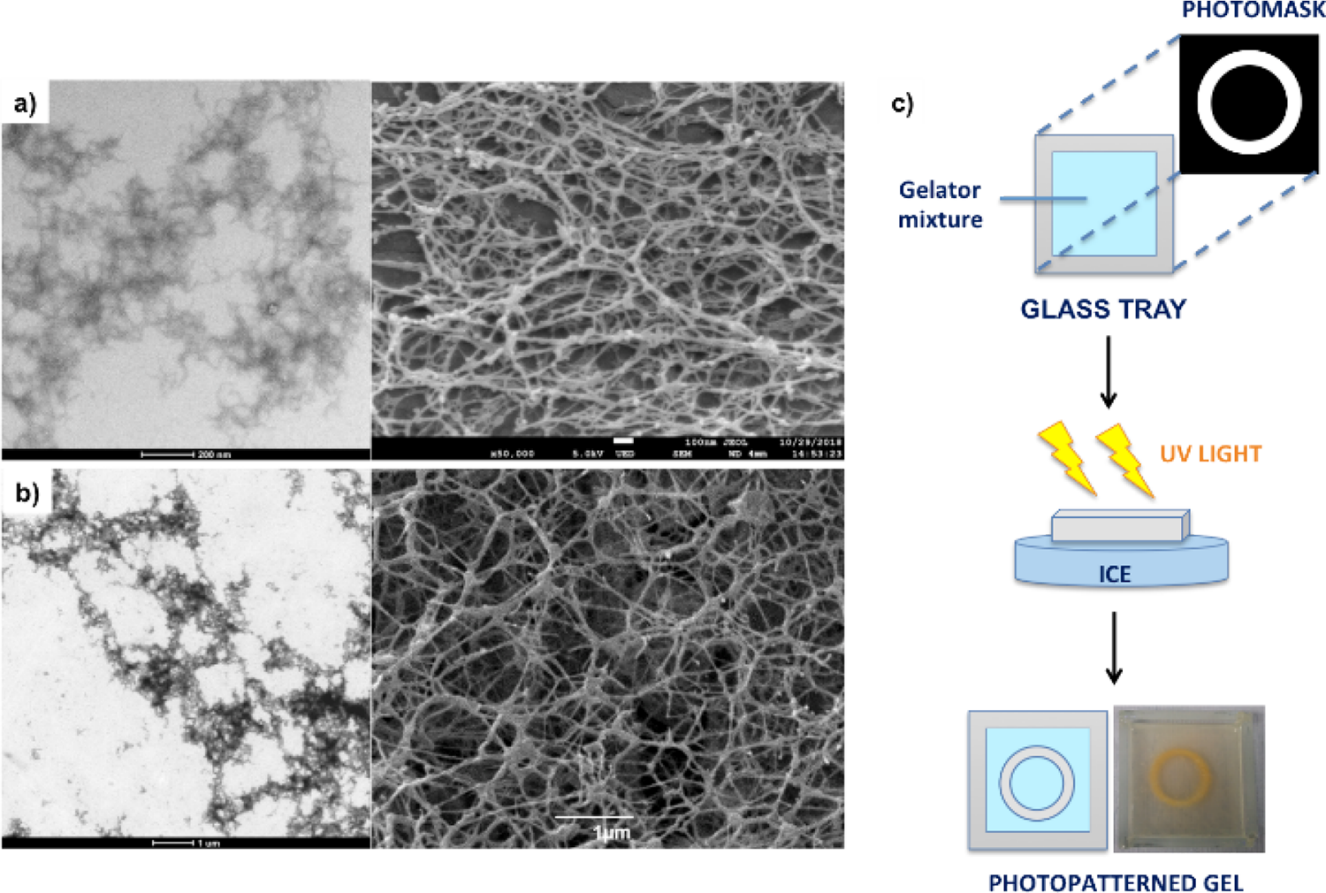
(a,b) TEM and SEM images of DBS-CONHNH_2_/alginate gels prepared using different pH activators for CaCO_3_: (a) GdL, scale bars of 200 (left) and 100 nm (right); (b) DPIN, scale bars of 1 μm. (c) Schematic representation of photopatterning using a mask with a circular pattern and a photographic image of a DBS-CONHNH_2_/alginate photopatterned gel, where the gel tray has dimensions of 5 cm × 5 cm and the ring has an outer diameter of ca. 2.20–2.25 cm and a width of 0.30–0.35 cm.

The morphology of the DBS-CONHNH_2_/alginate gel fibers obtained by photoactivation was analyzed by TEM and SEM, and it was broadly similar to the hybrid gel prepared in sample vials using GdL (Figure 2a,b and Figures S6 and S25). However, the fiber width appeared to be larger for the UV-triggered DPIN-activated gels (30–70 nm) compared with the gels prepared with GdL (10–30 nm; Figures S7 and S26). This might indicate a more rapid change in pH under photoirradiation conditions giving rise to a slightly less well-controlled self-assembly process and hence somewhat larger-diameter assemblies. This observation is consistent with the observation that the gel network is significantly less stiff when DPIN is used rather than GdL.

#### Preparation of Photopatterned Gels in Trays

As noted above, the key advantage of UV activation is the potential to induce gel patterning by controlling the parts of the gel that undergo photoirradiation. We therefore briefly explored the use of photopatterning through the application of a photomask with a desired pattern. We have previously demonstrated that DBS-based gels provide a good supportive network for gel-in-gel patterning, limiting diffusion and convection effects and allowing the fabrication of well-resolved patterned materials incorporating other gelators.^57−60^ We aimed to make use of these properties here and demonstrate this fabrication method-enabled patterning to also be achieved for hybrid LMWG/PG gels including calcium alginate.

DBS-CONHNH_2_/alginate photopatterned gels were prepared by combining DBS-CONHNH_2_ (0.3% wt/vol, 6.3 mM) with CaCO_3_ (0.15% wt/vol, 15 mM), DPIN (0.8% wt/vol, 23.3 mM), and sodium alginate (0.3% wt/vol). The mixture was heated until complete dissolution of the LMWG and then transferred to a 5 × 5 cm glass tray (Figure 2c). The sample was left undisturbed for 15 min to allow the initial formation of the thermally induced DBS-CONHNH_2_ network. A laser printed mask was then placed on top of the glass tray, and the gel was exposed to UV light for 2 h. To avoid disruption of gelation due to heating effects, ice was placed below the glass tray. After photoirradiation, the desired pattern formed by the cross-linked alginate was clearly visible within the DBS-CONHNH_2_ gel (Figure 2c)—the gel becomes opaque because of the formation of iodobenzene as a byproduct.^59^ It is evident that this occurred with good resolution. The width of the gel ring has an external diameter of ca. 2.20–2.25 cm and a width of ca. 0.30–0.35 cm, in good agreement with the mask that was used and indicative of an effective patterning process.

**Figure 3:**
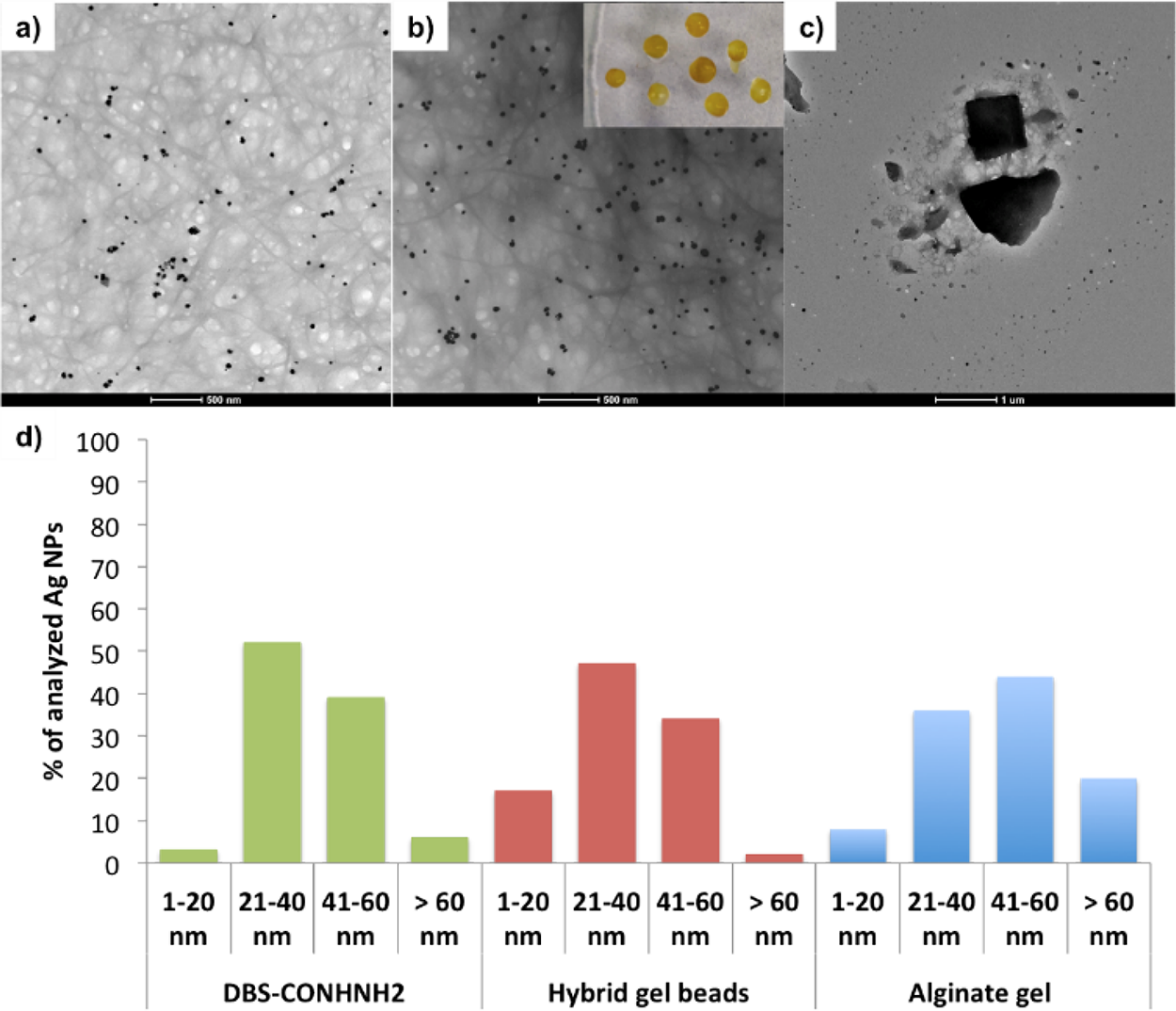
(a–c) TEM images of the DBS-CONHNH_2_ gel ((a) scale bar of 500 nm), DBS-CONHNH_2_/alginate beads ((b) scale bar of 500 nm), and the alginate gel ((c) scale bar of 1 μm) incorporating AgNPs. Scale bars: 500 nm (left and center) and 1 μm (right). (d) AgNP diameter size ranges in the DBS-CONHNH_2_ gel, DBS-CONHNH_2_/alginate beads, and the alginate gel.

### In Situ Formation of Ag Nanoparticles (NPs) in Hybrid Gel Beads

Having demonstrated that pH-mediated calcium carbonate dissolution could be harnessed to create shaped and patterned hybrid gels with DBS-CONHNH_2_, we then wanted to demonstrate that the LMWG retained its unique characteristics within this type of dual-network material. We therefore decided to induce the in situ formation of silver nanoparticles (AgNPs) within the gel beads. This exploits the reducing power of DBS-CONHNH_2_, which reduces Ag(I) to Ag(0) when exposed to silver salt solutions, leading to the formation of AgNPs.^25^ We previously studied the in situ formation of AgNPs in core–shell DBS-CONHNH_2_/alginate gel beads and reported on their antibacterial properties.^21^ The gels here show different spatial arrangements of the two gelators (i.e., interpenetrating rather than core–shell). We were, therefore, interested in confirming that, despite the different architecture, DBS-CONHNH_2_ could maintain its function.

To test the processes and their biocompatibility, DBS-CONHNH_2_/alginate gel beads were prepared using our standard conditions by combining DBS-CONHNH_2_ (0.3% wt/vol, 6.3 mM) with alginate (0.5% wt/vol), GdL (0.8% wt/vol), and CaCO_3_ (0.15% wt/vol) and compared to DBS-CONHNH_2_ and alginate gels alone prepared in sample vials. To remove residual ions, the beads were washed multiple times with water. AgNP formation was then induced by immersing the gel beads in a solution of AgNO_3_ (10 mM, 1 or 3 mL) for 72 h. The formation of AgNPs was confirmed by the color change of the beads (from white to orange; Figure 3b) and by TEM, which clearly showed the presence of AgNPs dispersed between gel fibers with average diameters of 20–60 nm (Figure 3b,d and Figure S32), similar to those formed in the DBS-CONHNH_2_ gel (Figure 3a,d and Figure S31). Some sorts of AgNPs were also formed in the alginate gels (Figure 3c,d and Figure S33);^21^ however, these aggregates were not uniformly distributed and showed very variable sizes, including the presence of large, poorly defined aggregates.

The maximum amount of Ag(I) incorporated into the gel beads was quantified by precipitation titration of NaCl, in the presence of K_2_CrO_4_ as an indicator. Each gel bead (20 μL volume) could incorporate ca. 0.3 μmol of Ag(I), corresponding to ca. 15 μmol of Ag(I)/mL of gel (Table S5). This is very similar to the Ag(I) uptake in the DBS-CONHNH_2_ gels (16.7 μmol of Ag(I)/mL of gel; Table S4), thus confirming that the LMWG retains its function within the hybrid gel beads. There is 6.3 μmol of DBSCONHNH_2_/mL of gel, and with each molecule containing two acyl hydrazide groups, this gives an effective acyl hydrazide concentration of 12.6 μmol/mL of gel, consistent with our hypothesis that the acyl hydrazide group is responsible for the in situ reduction process.^21,25^

The mechanical properties of the AgNP hybrid gels prepared in sample vials (0.3% DBS-CONHNH_2_, 0.5% alginate, 0.15% CaCO_3_, and 0.8% GdL) were studied by oscillatory rheology. Overall, the hybrid gels loaded with AgNPs (10 or 30 μmol/mL of gel) showed significantly lower elastic moduli (*G*′ = 1320 or 584 Pa, respectively) than the unloaded gels (*G*′ = 4090 Pa; Table S8 and Figures S36 and S37). This was also observed for the DBS-CONHNH_2_ gels (Table S8 and Figures S34 and S35) and is probably due to AgNP-induced disruption of the interactions between fibers within the gel network. Interestingly, DBS-CONHNH_2_/alginate gels cross-linked with CaCl_2_ showed an increase in elastic moduli for increasing AgNP loadings.^21^ We hypothesize that the different trend in the CaCO_3_ gels’ mechanical properties may be due to the formation of a Ag_2_CO_3_ precipitate by the reaction of Ag^+^ ions with any residual CaCO_3_, which could be disruptive to the supramolecular interactions.

### Stem Cell Growth on Gel Beads and Antibacterial Activity

Hydrogels are interesting materials for stem cell growth in regenerative medicine.^65−67^ Furthermore, the presence of AgNPs is known to be beneficial to osteogenic differentiation of human mesenchymal stem cells.^68−71^ Hydrogels incorporating AgNPs may therefore be promising materials in bone tissue engineering and for the fabrication of orthopedic implants.

We therefore explored whether the DBS-CONHNH_2_/alginate hybrid gel beads could support human stem cell growth. Preliminary cytotoxicity and viability experiments were performed on a human mesenchymal stem cell line (Y201)^72^ using different AgNP loadings. The DBS-CONHNH_2_/alginate hybrid gels and alginate gels for cytotoxicity testing were prepared in a 48-well plate and loaded with 12.5 μM or 10 mM AgNO_3_ (0.0125 or 10.0 μmol of AgNO_3_/mL of gel, respectively). The samples were then transferred in the middle of a 6-well plate, where the cells were seeded. Due to their fragility, DBS-CONHNH_2_ gels in the absence of calcium alginate could not be transferred from one plate to another; therefore, these gels were prepared directly on the 6-well plates using bottomless vials, which did not allow loading of the gels with AgNO_3_. For this reason, this test was not carried out on DBS-CONHNH_2_ gels incorporating AgNPs. After 48 h, the cells were stained with crystal violet and imaged. Pleasingly, the gels without AgNPs and those incorporating a modest Ag loading concentration of 12.5 μM did not show any “zone of inhibition” of cell growth (Figures S40 and S41), indicating their biocompatibility. However, the gels incorporating a high Ag concentration (10.0 mM AgNO_3_) showed a rather large empty area around them (2.90–3.60 mm; Figure S41), indicating the toxicity of these materials to stem cells. This is in line with previous studies showing that high concentrations of Ag^+^ ions can affect mesenchymal stem cell survival and function in vitro and in vivo.^73,74^

To obtain further preliminary data on biocompatibility and explore a range of nontoxic AgNP concentrations, we performed an Alamar Blue assay on Y201 cells grown on gels with different AgNP loadings and control gels without AgNPs.

DBS-CONHNH_2_ and alginate gels were prepared directly on 96-well plates, whereas the hybrid gel beads were prepared by emulsion and subsequently transferred to the 96-well plates. To make sure that the cells were adhering to the gels rather than the plate surface, we used nonadherent plates. The gels were loaded with different AgNO_3_ loadings (0.00625, 0.0125, 0.05, 0.1, 1.0, and 10 μmol of AgNO_3_/mL of gel, i.e., 6.25 μM, 12.5 μM, 50 μM, 100 μM, 1 mM, and 10 mM AgNO_3_, respectively), and cell metabolic activity was monitored over 10 days (days 0, 3, 6, and 10).

**Figure 4:**
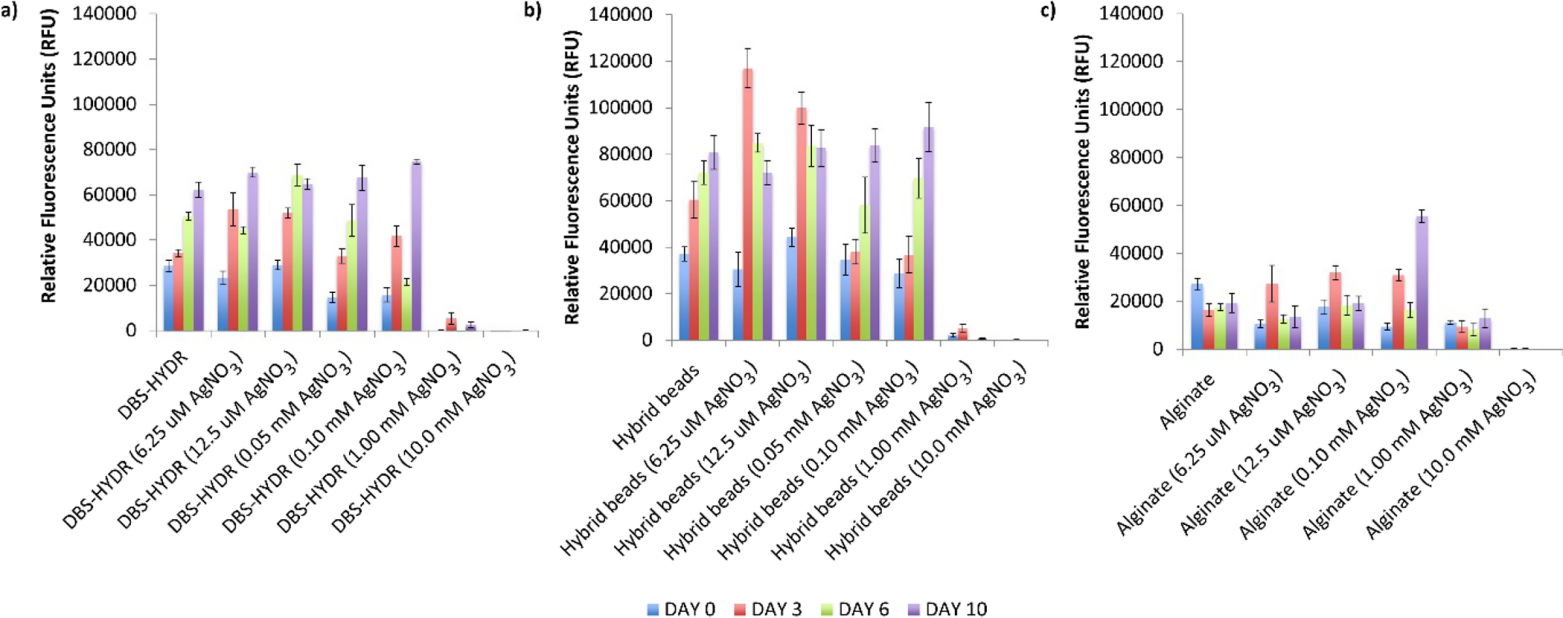
Alamar Blue viability assay results at days 0, 3, 6, and 10 for (a) DBS-CONHNH_2_ (DBS-HYDR), (b) DBS-CONHNH_2_/alginate (hybrid) beads, and (c) alginate gels loaded with AgNPs (0.00625, 0.0125, 0.05, 0.1, 1.0, and 10 μM AgNO_3_/mL of gel, i.e., 6.25 μM, 12.5 μM, 50 μM, 100 μM, 1 mM, and 10 mM AgNO_3_, respectively) and control gels without AgNPs.

Pleasingly, the results obtained showed that the cells were metabolically active in the gels without AgNPs and in those loaded with 6.25–100 μM AgNO_3_ (Figure 4). As expected, higher AgNO_3_ loadings (1.0 and 10 mM AgNO_3_) were toxic across the different gel types tested. Compared to the standard DBS-CONHNH_2_ gels, the hybrid gel beads showed higher fluorescence values over 10 days, indicative of a higher cell metabolic activity, which can be related to a higher number of cells. This is probably due to the higher surface area of the gel beads available for cell anchorage and penetration inside the gels. Overall, the alginate-only gels displayed significantly lower fluorescence values over the 10 days of the test, which indicates that they are less effective in supporting stem cell growth. It is important to note that soaking alginate-only gels in AgNO_3_ is not an effective methodology for inducing AgNP formation, which leads instead (as described above) to the formation of bigger, nonuniformly distributed metal aggregates. The lower cell viability may be related to these aggregates. The use of reducing agents, such as NaBH_4_, would be a more efficient strategy to form AgNPs in such gels.^75−78^ However, all gels were simply exposed to AgNO_3_ to compare them under the same conditions.

**Figure 5:**
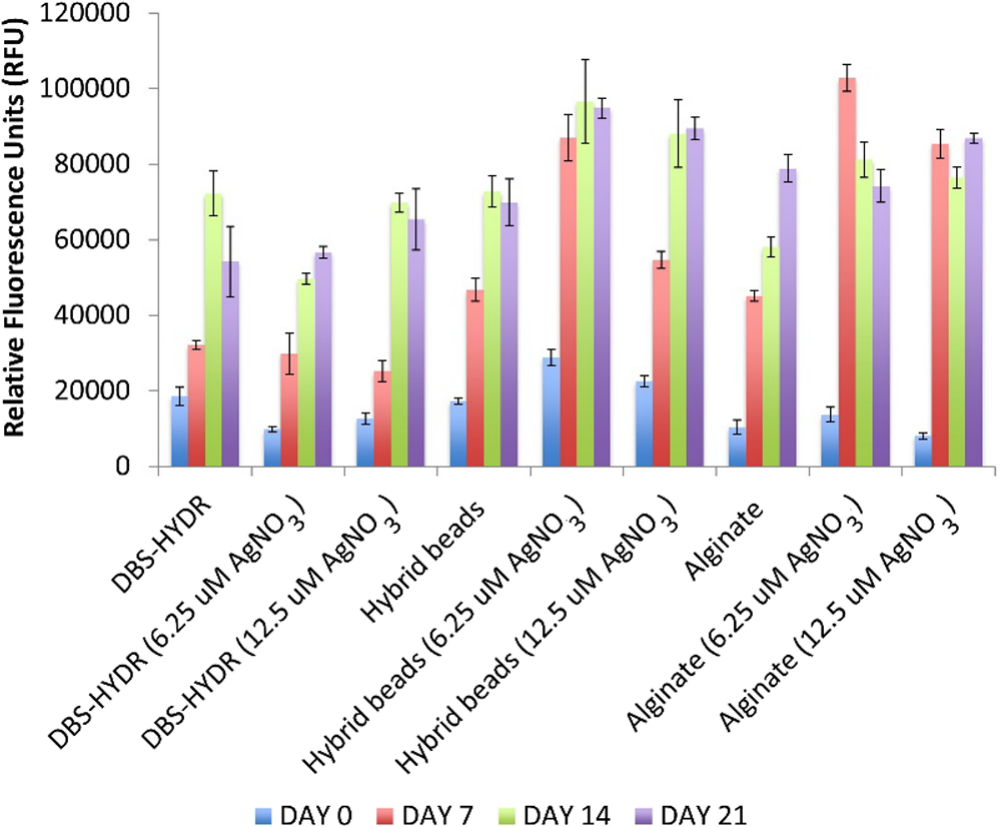
Alamar Blue viability assay results at days 0, 7, 14, and 21 for DBS-CONHNH_2_ (DBS-HYDR), DBS-CONHNH_2_/alginate (hybrid) beads, and alginate gels loaded with AgNPs (0.00625 and 0.0125 μmol of AgNO_3_/mL of gel) and control gels without AgNPs.

Finally, to verify if the gels could support cell growth over a longer period of time, a viability test was performed over 21 days (days 0, 7, 14, and 21) on the gels loaded with the optimized lower AgNO_3_ loadings (6.25 and 12.5 μM AgNO_3_, Figure 5) and control gels without AgNPs. Pleasingly, the results showed that the cells were metabolically active in all gels for the whole duration of the study, with the hybrid beads loaded with AgNPs being most effective at day 21.

It is well-known that AgNPs have antimicrobial properties,^79−84^ and therefore, we were interested to determine whether these AgNP-loaded gels had antibacterial activity. It is known from the literature that AgNPs are a double-edged sword, with antibacterial properties but also having the potential for toxicity against mammalian cells.^85,86^ However, AgNPs can exhibit antibacterial properties at concentrations as low as 1 mg/L (ca. 9 μM), comparable with the lowest concentrations used in this study (6.25 and 12.5 μM), at which our stem cells were completely unaffected; indeed, we saw no evidence of toxicity up to an Ag concentration of ca. 11 mg/L (100 μM). We were therefore interested by the concept that these gels may have antibacterial applications.

**Figure 6:**
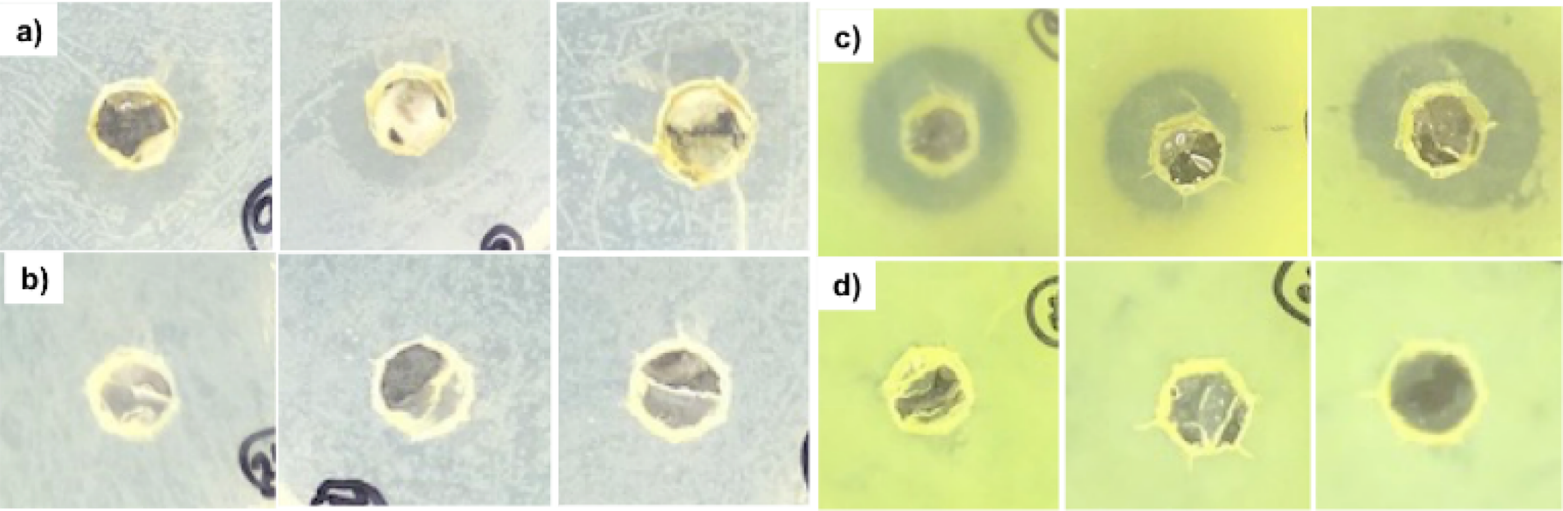
Photographic images of the disc diffusion assay. (Left) Vancomycin-resistant *Enterococcus faecium* (VRE). (Right) *Pseudomonas aeruginosa* (PA14). (a,c) DBS-CONHNH_2_/alginate gels loaded with AgNPs; the dark rings indicate the zone of inhibition. (b,d) DBS-CONHNH_2_/alginate control gels without AgNPs. Water, kanamycin, and vancomycin controls are reported in the Supporting Information (Figures S41 and S42). Images are 10 mm × 10 mm.

We performed a preliminary disc diffusion assay on two different bacterial strains using gels with high AgNP loadings (10 mM AgNO_3_). This study (see the Supporting Information for details) demonstrated that the growth of a vancomycin-resistant *Enterococcus faecium* (VRE), a Gram-positive bacterium, and *Pseudomonas aeruginosa* (PA14), a Gram-negative bacterium, was inhibited by the AgNP-loaded gels (Figure 6a,c, Figure S44, and Table S9). No zone of inhibition was observed for the gels that did not incorporate AgNPs (Figure 6b,d, Figure S46, and Table S9) or the controls (Figures S42 and S43).

We were interested to know whether silver ions were released during the bacterial growth assay. There has been debate over the antimicrobial mode of action of AgNPs, with Ag(I) ions having antimicrobial properties but AgNPs having distinct mechanisms of action.^87−89^ We therefore suspended the beads in water and analyzed the solution for Ag(I) (Tables S6 and S7). A small amount of Ag(I) (ca. 20%) was initially released from the gel, but after 30 min, no further silver ions were released (Figure S30). This small amount of initial release would suggest that the AgNPs are not releasing silver ions but rather that a small amount of Ag^+^ remains associated with the gel and is rapidly released. This leads us to suggest that the longer-term antimicrobial activity associated with these gel beads is more likely associated with reactive oxygen species produced by the AgNPs embedded in the gels, although this requires further study.

Overall, this study confirms that these AgNP-loaded gel beads are active against some drug-resistant bacteria at relatively high Ag loadings and may have potential antibacterial uses. However, it is important to note that more detailed studies will be required to determine whether conditions can be found for these materials under which both stem cell growth and antibacterial activity can be achieved at the same time. If so, then this would open up potential applications of these AgNP-loaded shapeable biomaterials as effective fillers to facilitate bone regeneration while simultaneously preventing opportunistic infections.^90−92^

## Conclusions

In conclusion, we report an alternative way to fabricate DBS-CONHNH_2_/alginate gels by pH-triggered alginate cross-linking in the presence of CaCO_3_. This was achieved using GdL as the acid source within preformed LMWG template beads forming well-defined beads with interpenetrating LMWG and PG networks. Alternatively, by substituting GdL with the photoacid generator DPIN, alginate gelation can be induced by photoirradiation in a DBS-CONHNH_2_ gel tray support, allowing spatially resolved photopatterning of the gel—a rare report of photopatterning a multidomain LMWG/PG material. Importantly, the LMWG not only acts as a supporting scaffold for alginate gelation but also keeps its function of reducing precious metals within the hybrid gels, as demonstrated by the in situ formation of AgNPs on simple exposure of the gels to a solution of Ag(I).

Preliminary biological testing indicated that human mesenchymal stem cells can survive and thrive in the gels for long periods of time (i.e., at least 21 days). Furthermore, at high silver loadings, the AgNP-loaded gel beads exhibited antibacterial properties against drug-resistant bacteria. We suggest that our DBS-CONHNH_2_/alginate beads may be promising materials either in regenerative medicine or antibacterial applications. With further optimization, the two activities of these gels may also be combined, giving them uses in orthopedic applications where tissue growth is desired alongside an ability to prevent opportunistic infections. Future studies will explore cell function in detail and osteogenic activity of cells grown on AgNP gel beads, as well as their antimicrobial properties in a relevant setting. In addition, we are testing AgNP formation in injectable DBS-CONHNH_2_/alginate microbeads^22^ and exploring their potential applications in wound healing.

## Supporting Information Available

Full details of gel fabrication methods and characterization data, biological assay methods, and additional data (PDF)
