# Supplementary material for: Shaping and Patterning Supramolecular Materials—Stem Cell-Compatible Dual-Network Hybrid Gels Loaded with Silver Nanoparticles
Source: ACS Biomater Sci Eng. 2022 Apr 2;8(5):1829–40. doi: 10.1021/acsbiomaterials.1c01560 (PMC9092345; doi:10.1021/acsbiomaterials.1c01560)
Supplement: Supplementary file 1 — ab1c01560_si_001.pdf [file ab1c01560_si_001.pdf]

Shaping and Patterning Supramolecular Materials – Stem Cell Compatible Dual-Network Hybrid Gels Loaded with Silver Nanoparticles

Carmen C. Piras,*,[a] Clare S. Mahon,[a] Paul G. Genever[b] and David K. Smith*,[a]

a: Department of Chemistry, University of York, Heslington, York, YO10 5DD, UK
b: Department of Biology, University of York, Heslington, York, YO10 5DD, UK
Email : david.smith@york.ac.uk

S1 General experimental methods

S2 Preparation and characterisation of DBS-CONHNH$_2$, DBS-CONHNH$_2$/alginate and alginate gels cross-linked with $CaCO_3$ and Glucono-δ-Lactone (GdL)
S2.1 Gel preparation
S2.2 pH studies
S2.3 NMR assay
S2.4 Infrared (IR) spectroscopy
S2.5 Optical microscopy
S2.6 Transmission and Scanning Electron Microscopy (TEM and SEM)
S2.7 Thermal stability studies
S2.8 Rheology

S3 Preparation and characterisation of UV responsive DBS-CONHNH$_2$/alginate and alginate gels
S3.1 Gel preparation and photopatterning
S3.2 NMR assay
S3.3 Transmission and Scanning Electron Microscopy (TEM and SEM)
S3.4 Thermal stability studies
S3.5 Rheology

S4 Preparation and characterisation of DBS-CONHNH$_2$, DBS-CONHNH$_2$/alginate and alginate gels loaded with Ag nanoparticles (NPs)
S4.1 *In situ* formation of Ag NPs
S4.2 Uptake of Ag(I)
S4.3 Release of Ag(I) from DBS-CONHNH$_2$/alginate gel beads loaded with Ag NPs
S4.4 Transmission Electron Microscopy (TEM)
S4.5 Rheology

S5 Biological studies
S5.1 Cell line
S5.2 Cytotoxicity assays
S5.3 Viability assay

S6 Disc diffusion assays

S7 References

S1 General Experimental Methods

All compounds used in synthesis and analysis were purchased from standard commercial suppliers and used as received. The alginate employed in all the experiments was bought from Sigma Aldrich as sodium salt (2% viscosity). The synthesis of DBS-CONHNH$_2$ was performed in good yields applying previously reported methods.[1, 2] ^{1}H NMR spectra were recorded using a Jeol 400 spectrometer (^{1}H 400 MHz). Samples were prepared in DMSO-d$_6$ or D_2O and chemical shifts (δ) are reported in parts per million (ppm). IR spectra of xerogels were recorded on a PerkinElmer Spectrum Two FT-IR spectrometer. Optical microscopy images were obtained using a Zeiss Axiocam camera on a Zeiss stereo microscope. SEM images were taken using a JEOL JSM-7600F field emission SEM. TEM images were obtained on a FEI Tecnai 12 G^2 fitted with a CCD camera. Fibre sizes and gel bead diameters were measured using the *ImageJ* software. T_{gel} values were obtained using a high precision thermoregulated oil bath using the tube inversion method and were recorded in triplicate. Rheology was measured on a Malvern Instruments Kinexus Pro+ Rheometer fitted with a 20 mm parallel plate geometry. A high-powered UV lamp (λ = 315-405 nm) was used for activation of the photoacid generator diphenyliodonium nitrate (DPIN). Fluorescence measurements for the cell viability assay were performed using a BMG Labtech Clariostar Plate Reader.

S2 Preparation and characterisation of DBS-CONHNH$_2$, DBS-CONHNH$_2$/alginate and alginate gels cross-linked with $CaCO_3$ and Glucono-δ-Lactone (GdL)

S2.1 Gel preparation

S2.1.1 Preparation of DBS-CONHNH$_2$ gels. DBS-CONHNH$_2$ (0.3 or 0.4% wt/vol) was suspended in water (1 mL). The suspension was sonicated to help the dispersion of the solid particles and then heated until complete dissolution of the compound. The sample was left undisturbed to cool, allowing gel formation in few minutes.

S2.1.2 Preparation of DBS-CONHNH$_2$/alginate multicomponent gel beads. DBS-CONHNH$_2$ (0.3% wt/vol in 1 mL final total volume) and $CaCO_3$ (0.15% wt/vol in 1 mL final total volume) were suspended in water (0.5 mL) and sonicated to help the dispersion of the solid particles. An aqueous alginate solution (1.0% wt/vol - 0.5 mL) and GdL (0.8% wt/vol in 1 mL final total volume) were then added. The amount of alginate and water was adjusted depending on the desired final concentration of the polymer in the different experiments. The resulting suspension was heated until complete dissolution of the DBS-CONHNH$_2$ (insoluble $CaCO_3$ remained) The hot solution was then added dropwise (20 μL/drop) to paraffin oil (c.a. 50 mL). The droplets were left undisturbed overnight to allow gel formation. After this time, the gel beads were collected with a spatula and, to remove residual paraffin oil, they were immersed in petroleum ether (30 mL, 30 mins), then EtOH (30 mL, 30 mins) and, finally, water (30 mL, 30 mins). When necessary, the washings were performed multiple times. The gel beads were then stored in water.

S2.1.3 Preparation of DBS-CONHNH$_2$/alginate gels in sample vials using GdL as a pH activator (for pH, IR, thermal stability and rheology studies). DBS-CONHNH$_2$ (0.3% wt/vol in 1 mL final total volume) and $CaCO_3$ (0.15% wt/vol in 1 mL final total volume, unless otherwise specified) were suspended in water (0.5 mL) and sonicated to help the dispersion of the solid particles. An aqueous alginate solution (1.0% wt/vol - 0.5 mL) was then added. The amount of alginate and water was adjusted depending on the desired final concentration of the polymer in the different experiments. The resulting suspension was heated until

complete dissolution of the DBS-CONHNH$_2$ (insoluble $CaCO_3$ remained) and then transferred to a new sample vial containing GdL (0.8% wt/vol in 1 mL final total volume, unless otherwise specified). The sample was left undisturbed overnight to allow gel formation.

S2.1.4 Preparation of alginate gels using GdL as a pH activator. Alginate gels were prepared by adding $CaCO_3$ (0.15% wt/vol in 1 mL final total volume, unless otherwise specified) to an aqueous alginate solution (0.4-1.3% wt/vol). The suspension was sonicated to help the dispersion of the $CaCO_3$ solid particles and GdL (0.8% wt/vol in 1 mL final total volume, unless otherwise specified) was then added. The sample was left undisturbed overnight to allow gel formation.

S2.2 pH studies

The pH of the DBS-CONHNH$_2$/alginate hybrid gels and alginate gels was monitored over time at regular time intervals during gel formation. The gels were prepared in sample vials as described in Sections S2.1.3 and S2.1.4 using variable alginate concentrations (0.1-0.8% wt/vol) in the presence of $CaCO_3$ (0.15% wt/vol) and GdL (0.8% wt/vol).

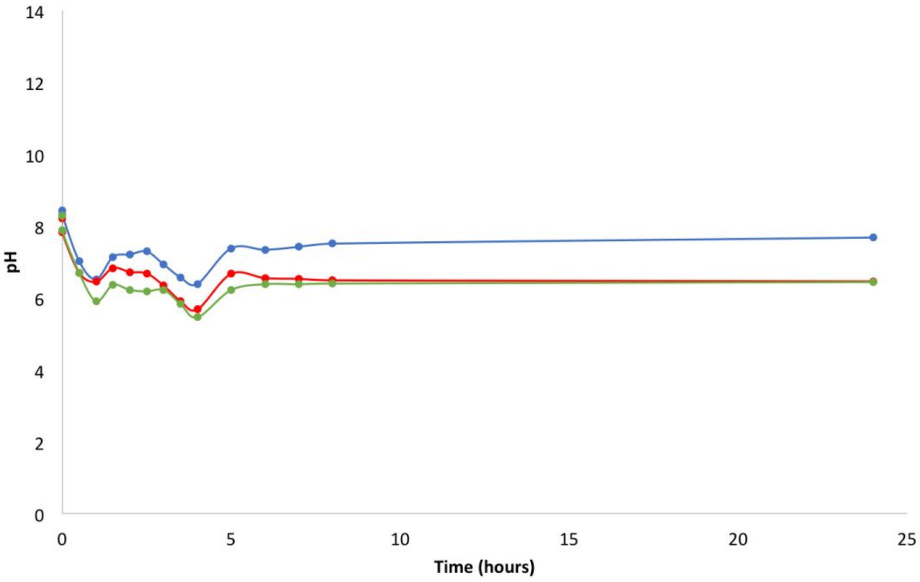

Figure S1. pH changes over time during the formation of the DBS-CONHNH$_2$/alginate hybrid gel ($CaCO_3$ - 0.15% wt/vol and GdL - 0.8% wt/vol) containing: 0.3% wt/vol of DBS-CONHNH$_2$ and 0.1% wt/vol alginate (blue line), 0.3% wt/vol alginate (red line) or 0.5% wt/vol alginate (green line).

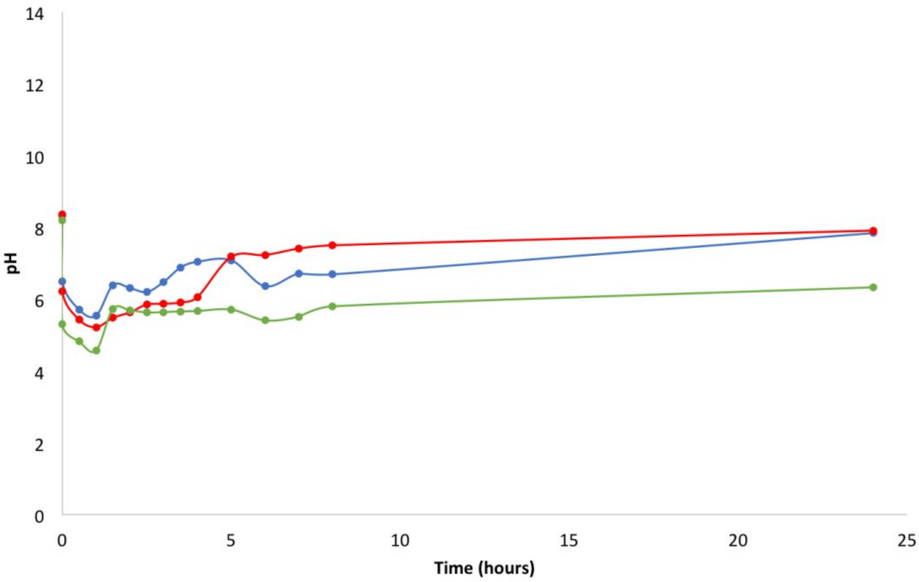

Figure S2. pH changes over time during the formation of alginate gel ($CaCO_3$ - 0.15% wt/vol and GdL - 0.8% wt/vol) prepared using an alginate concentration of 0.4% wt/vol (blue line), 0.6% wt/vol (red line) or 0.8% wt/vol (green line).

S2.3 NMR assays

S2.3.1 *Self-assembled state of DBS-CONHNH$_2$/alginate gel beads.* ^{1}H NMR was employed to validate the efficacy of the gel preparation method and to confirm that the two gelators were in a self-assembled state. The gel beads used for this experiment were prepared combining DBS-CONHNH$_2$ (0.3 % wt/vol) and alginate (0.5 % wt/vol) by the emulsion method described in Section S2.1.2. Five gel beads were isolated and transferred into a NMR tube in D_2O (0.7 mL). DMSO (1.4 μL) was added as an internal standard. The gel beads were then analysed by ^{1}H NMR. The lack of DBS-CONHNH$_2$ aromatic signals confirms its self-assembled state. Since the alginate signals overlap with those of GdL, it is not possible to drag any conclusion for the PG.

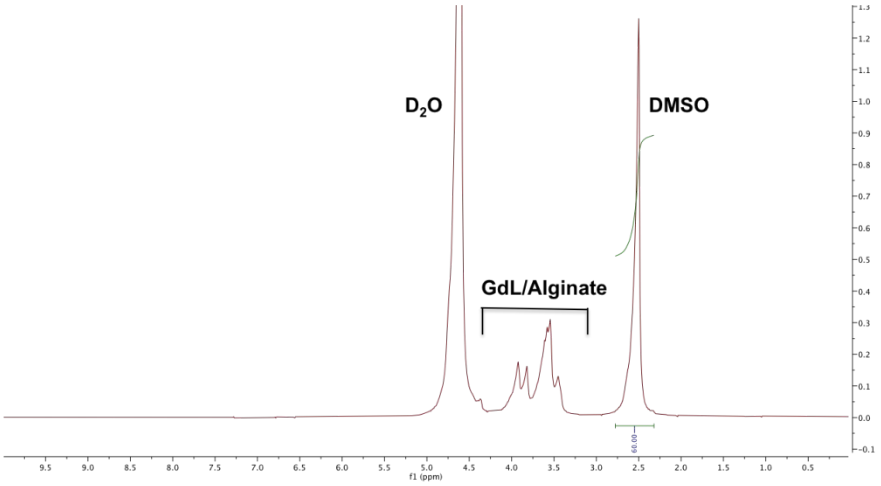

Figure S3. ^{1}H NMR of five DBS-CONHNH$_2$/alginate gel beads.

S2.3.2 *Quantification of DBS-CONHNH$_2$ incorporated into ten DBS-CONHNH$_2$/alginate gel beads.* ^{1}H NMR was employed to calculate the exact amount of DBS-CONHNH$_2$ incorporated into the DBS-CONHNH$_2$/alginate gel beads prepared by emulsion. The gel beads used for this experiment were prepared

combining DBS-CONHNH$_2$ (0.3 % wt/vol) and alginate (0.5 % wt/vol) by the emulsion method described in Section S2.1.2. Ten gel beads were isolated and dried under high vacuum. The resulting solid was dissolved in DMSO-d$_6$ (0.7 mL), and acetonitrile (1.4 μL) was added as an internal standard. To make sure that all the DBS-CONHNH$_2$ was dissolved, the sample was ground and then sonicated for 30 min. The ^{1}H NMR spectrum was recorded and the concentration of the LMWG calculated by comparison of the integrals of relevant peaks (DBS-CONHNH$_2$ aromatic peaks δ = 7.53 and 7.83 ppm) to that of acetonitrile (δ = 2.09 ppm). To ensure the results were reproducible, this experiment was performed on two different batches of gel beads. It is noted that due to the low solubility of alginate in DMSO-d$_6$, the alginate peaks were not visible.

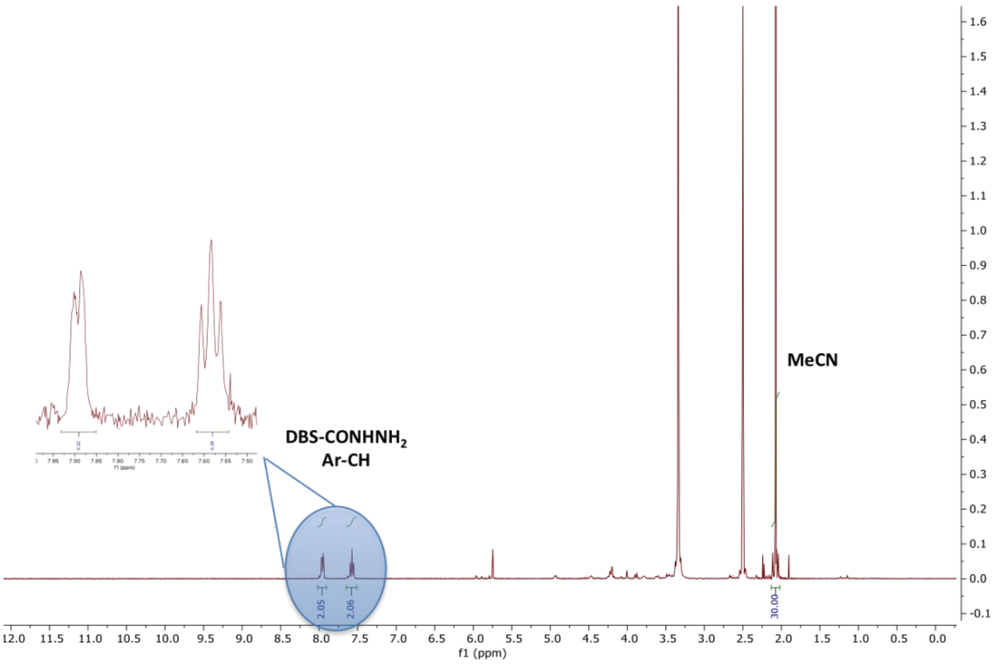

Figure S4. ^{1}H NMR of DBS-CONHNH$_2$ incorporated into 10 DBS-CONHNH$_2$/alginate multicomponent gel beads prepared using 0.3% wt/vol DBS-CONHNH$_2$ and 0.5% wt/vol alginate.

S2.4 Infrared (IR) spectroscopy

Xerogel samples for infrared were prepared in sample vials as described in Section S2.1.3 adding HCl (1M, 15 μL) instead of GdL. The solvent was then removed from the gels under high vacuum. A small amount of the resulting powder was placed into the infrared spectrophotometer and the spectra recorded in the range of 450-4000 cm^{-1}.

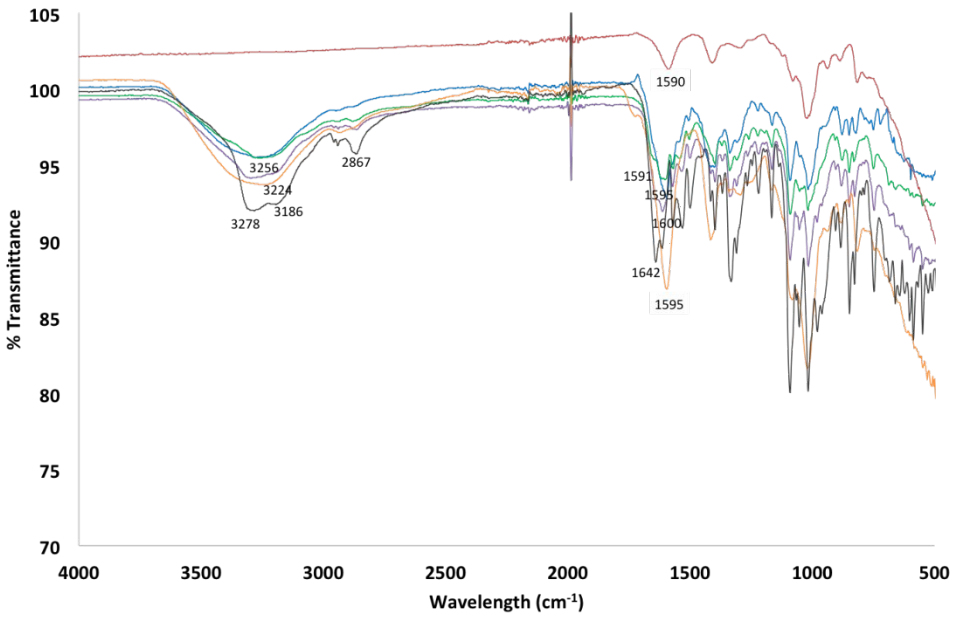

Figure S5. IR spectra of xerogels obtained from DBS-CONHNH$_2$ gel (0.4% wt/vol, black line), alginate gel (0.8% wt/vol, red line) and DBS-CONHNH$_2$/alginate gel containing 0.3% wt/vol of DBS-CONHNH$_2$ and 0.1% wt/vol alginate (purple line), 0.3% wt/vol alginate (blue line), 0.5% wt/vol alginate (orange line) and 1.0% wt/vol alginate (green line).

S2.5 Optical microscopy

Optical microscopy images were collected on a Zeiss stereo microscope. The gel beads were dehydrated through an ethanol series, then embedded in LR white resin. Sections were 1 μm thick. Once the section was dried on the slide, it was stained with Toluidine Blue (0.6% with 0.3% Na_2CO_3). All the gel beads were prepared in 20 μL volumes using 0.3% wt/vol of DBS-CONHNH$_2$ and 0.5% wt/vol of alginate.

S2.6 Transmission and Scanning Electron Microscopy (TEM and SEM)

S2.6.1 Preparation of samples for TEM. Samples for TEM were obtained by placing a small amount of each sample on a copper grid. The excess of sample was removed with filter paper and allowed to set for 5 min. A negative stain (1% uranyl acetate) was then added and the samples were left to rest for 30 min before taking the images. Fibre widths were determined using ImageJ software with the scale bar as a reference, and taking the average of 100 fibres diameters.

S2.6.2 Preparation of samples for SEM. Samples for SEM were obtained by freeze-drying the gels on copper shim pieces. The freeze-dried samples were then mounted on stubs and the images recorded. Alginate and hybrid beads were critical point dried (acetone and liquid CO_2) and mounted on stubs either whole, or halved using a razor blade. Mounted samples were sputter coated with Au/Pd. Fibre widths were determined using ImageJ software with the scale bar as a reference, and taking the average of 100 fibres diameters.

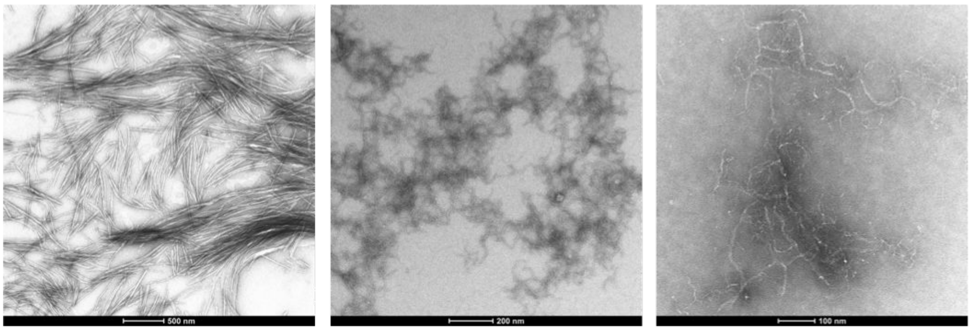

Figure S6. TEM images of DBS-CONHNH$_2$ gel (left), DBS-CONHNH$_2$/alginate hybrid gel (centre) and alginate gel (right) prepared using $CaCO_3$ as a cross-linker and GdL as a pH activator. Scale bars from left to right: 500, 200 and 100 nm.

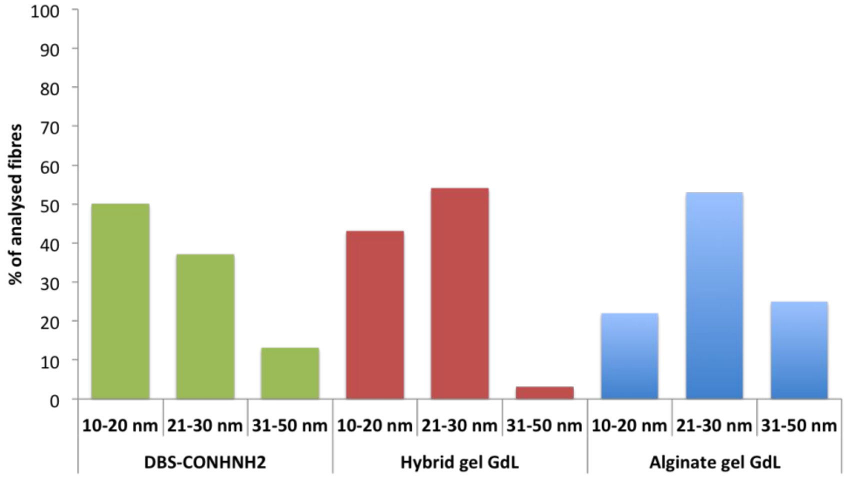

Figure S7. Fibre widths of DBS-CONHNH$_2$, DBS-CONHNH$_2$/alginate hybrid and alginate gels prepared using $CaCO_3$ as a cross-linker and GdL as a pH activator.

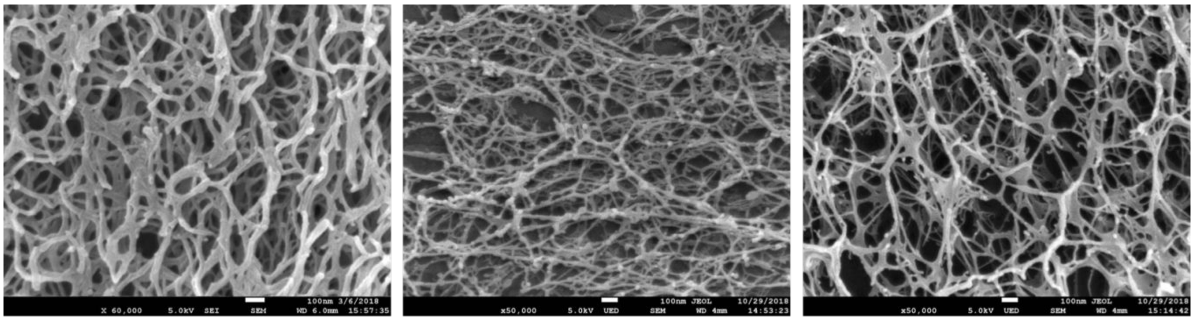

Figure S8. SEM images of DBS-CONHNH$_2$ gel (left), DBS-CONHNH$_2$/alginate hybrid gel (centre) and alginate gel (right) prepared using $CaCO_3$ as a cross-linker and GdL as a pH activator. Scale bars: 100 nm.

S2.7 Thermal stability studies

All the gels for T_{gel} determination were prepared as described in Section S2 in 2 mL vials (diameter: 1 cm, height: 4 cm). The gels were placed in a high precision thermoregulated oil bath with an initial temperature of 25°C. The temperature was increased by 1°C/ min until 100°C. Every minute the gels were checked by tube inversion method and T_{gel} was considered as the temperature at which the gel began to run down the

sides of the vial. These experiments were performed in triplicate to ensure reproducibility and the average is reported. Errors are estimated at ±2°C.

Table S1. T_{gel} values of gels formed by individual gelators and the DBS-CONHNH$_2$/alginate gel prepared using $CaCO_3$ as a cross-linker (0.15% wt/vol) and GdL (0.8% wt/vol) as a pH activator.

Gel (1 mL total volume)	Loading of DBS-CONHNH$_2$ (wt/vol)	Loading of ALGINATE (wt/vol)	T_{gel}
DBS-CONHNH$_2$	0.4%	-	86 °C
Alginate	-	0.4%	>100 °C
Alginate	-	0.6%	>100 °C
Alginate	-	0.8%	>100 °C
DBS-CONHNH$_2$/alginate hybrid gel	0.3%	0.1%	96 °C
DBS-CONHNH$_2$/alginate hybrid gel	0.3%	0.3%	>100 °C
DBS-CONHNH$_2$/alginate hybrid gel	0.3%	0.5%	>100 °C
DBS-CONHNH$_2$/alginate hybrid gel	0.3%	1.0%	>100 °C

S2.8 Rheology

Gel samples for rheology were prepared as described in Section S2 using bottomless vials as templates to obtain the intended gel dimensions. All the alginate and DBS-CONHNH$_2$/alginate hybrid gels were prepared using $CaCO_3$ (0.15% wt/vol) as a cross-linker and GdL (0.8% wt/vol) as a pH activator, unless otherwise specified. The measurements were carried out at 25°C using a 20 mm parallel plate and a gap of 2 mm. To avoid solvent evaporation and keep the sample hydrated, a solvent trap was used, and the internal atmosphere was kept saturated. Amplitude sweep experiments were performed in the range of 0.05-100% strain at a 1 Hz frequency to identify the linear viscoelastic region. Frequency sweep experiments were performed between 0.1 and 100 Hz using a shear strain of 0.25%. The measurements were repeated three times to ensure reproducibility and the average data are shown with standard deviation error bars.

Table S2. Rheological data as determined using oscillatory rheometry with a parallel plate geometry, for DBS-CONHNH$_2$ gels, calcium alginate gels, and hybrid gels formed by the combination of the two. Loadings are given in wt/vol, and the G'/G" crossover points refer to the % shear strain at which G"=G'.

Gel	Loading of LMWG	Loading of Alginate	Total Loading	Loading of $CaCO_3$	Loading of GdL	G' (Pa)	G'/G" Crossover
DBS-CONHNH$_2$	0.4%	-	0.4%	-	-	800	25.1%
Alginate	-	0.4%	0.4%	0.15%	0.8%	299	2.5%
Alginate	-	0.6%	0.6%	0.15%	0.8%	424	4.0%
Alginate	-	0.6%	0.6%	0.05%	0.8%	128	63.0%
Alginate	-	0.6%	0.6%	0.3%	0.8%	784	3.2%
Alginate	-	0.8%	0.8%	0.15%	0.8%	463	6.3%
Hybrid	0.3%	0.1%	0.4%	0.15%	0.8%	3360	18.6%
Hybrid	0.3%	0.3%	0.6%	0.15%	0.8%	3870	10.0%
Hybrid	0.3%	0.3%	0.6%	0.05%	0.8%	2110	8.0%
Hybrid	0.3%	0.3%	0.6%	0.3%	0.8%	3340	6.3%
Hybrid	0.3%	0.3%	0.6%	0.15%	1.0%	6190	6.7%
Hybrid	0.3%	0.3%	0.6%	0.15%	1.2%	6280	5.0%
Hybrid	0.3%	0.5%	0.8%	0.15%	0.8%	4090	6.6%
Hybrid	0.3%	1.0%	1.3%	0.15%	0.8%	4430	7.1%

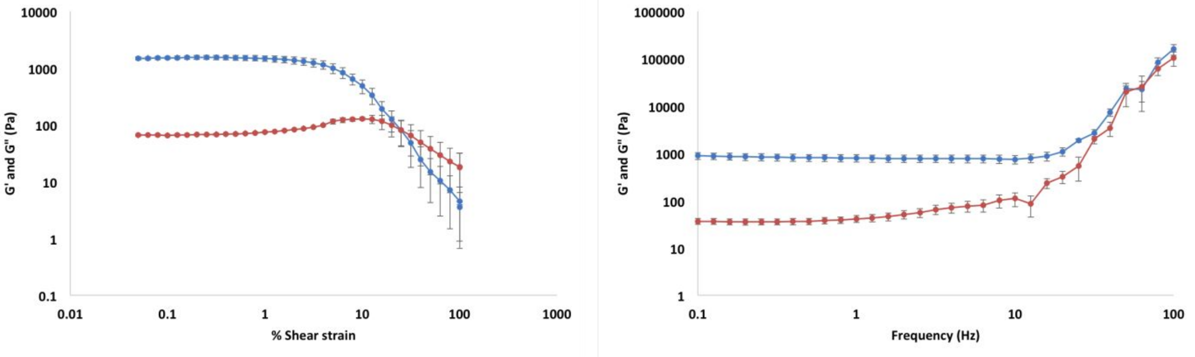

Figure S9. Elastic (G', blue circles) and viscous (G", red circles) moduli of DBS-CONHNH$_2$ hydrogel (0.4% wt/vol) with increasing shear strain (left) and frequency (right).

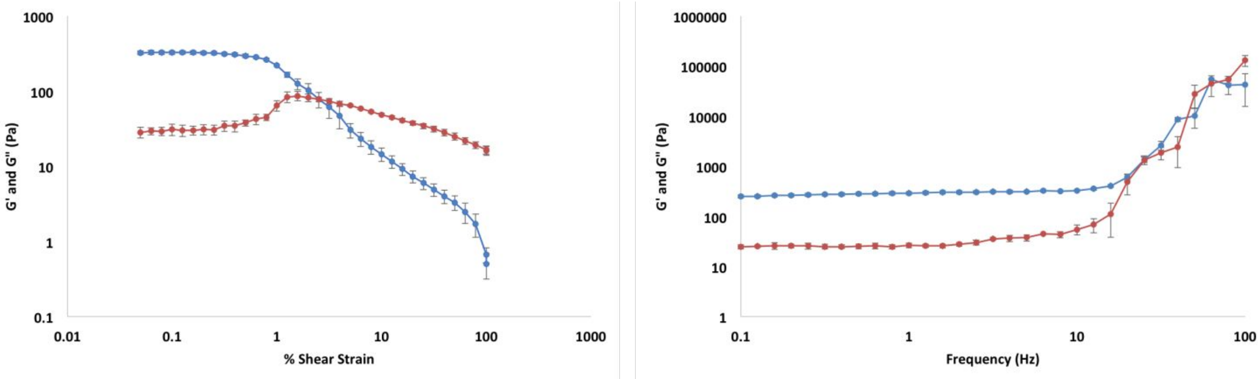

Figure S10. Elastic (G', blue circles) and viscous (G", red circles) moduli of alginate hydrogel (0.4% wt/vol) prepared with 0.15% wt/vol $CaCO_3$ and 0.8% wt/vol GdL with increasing shear strain (left) and frequency (right).

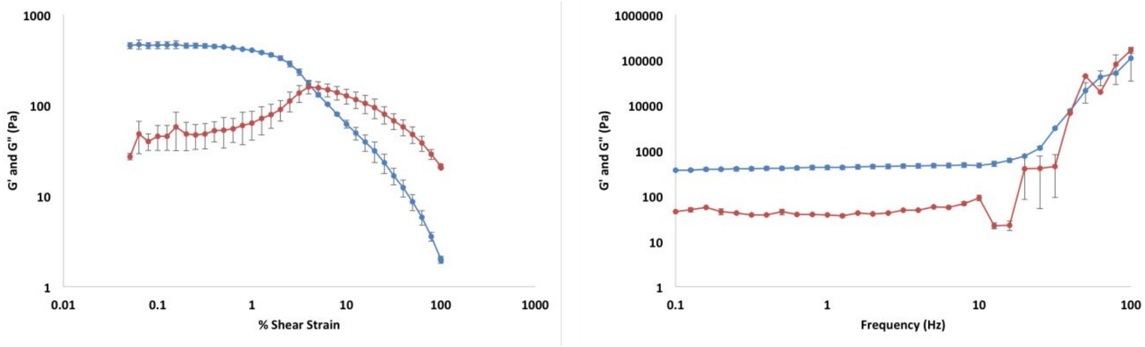

Figure S11. Elastic (G', blue circles) and viscous (G", red circles) moduli of alginate hydrogel (0.6% wt/vol) prepared with 0.15% wt/vol $CaCO_3$ and 0.8% wt/vol GdL with increasing shear strain (left) and frequency (right).

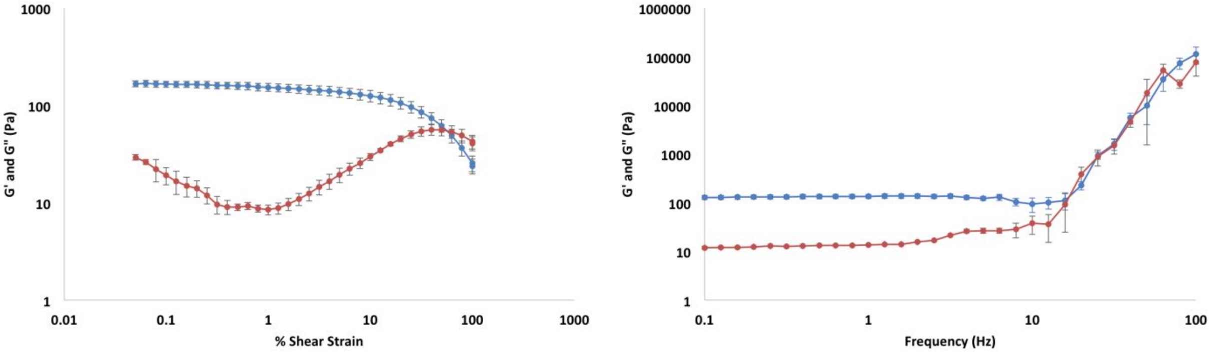

Figure S12. Elastic (G', blue circles) and viscous (G", red circles) moduli of alginate hydrogel (0.6% wt/vol) prepared with 0.05% wt/vol $CaCO_3$ and 0.8% wt/vol GdL with increasing shear strain (left) and frequency (right).

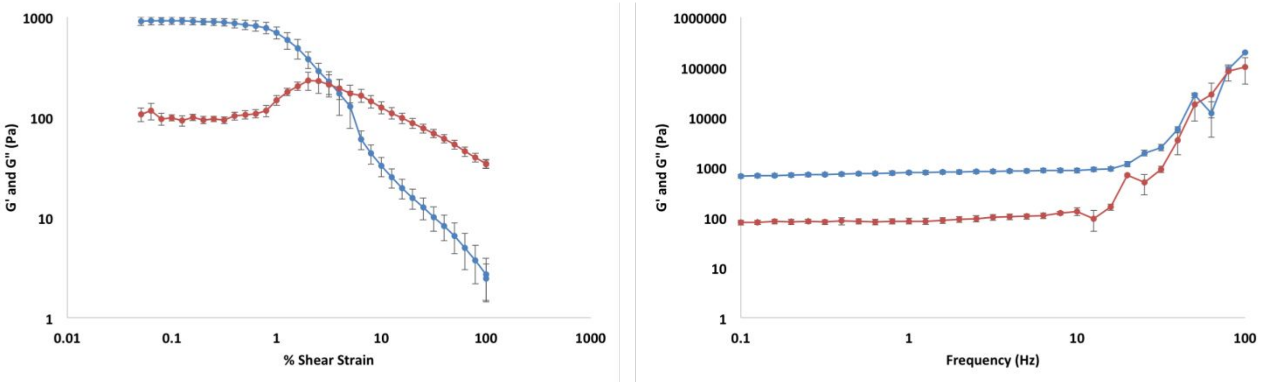

Figure S13. Elastic (G', blue circles) and viscous (G", red circles) moduli of alginate hydrogel (0.6% wt/vol) prepared with 0.3% wt/vol $CaCO_3$ and 0.8% wt/vol GdL with increasing shear strain (left) and frequency (right).

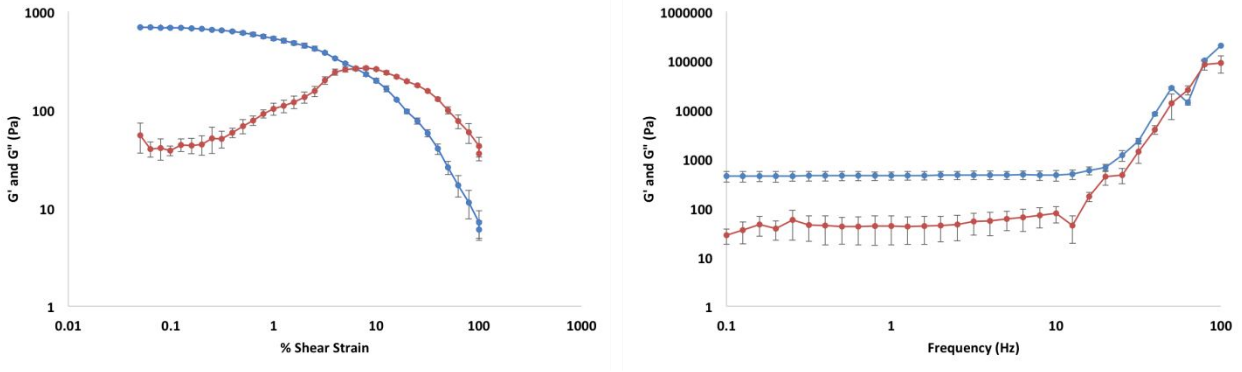

Figure S14. Elastic (G', blue circles) and viscous (G", red circles) moduli of alginate hydrogel (0.8% wt/vol) prepared with 0.15% wt/vol $CaCO_3$ and 0.8% wt/vol GdL with increasing shear strain (left) and frequency (right).

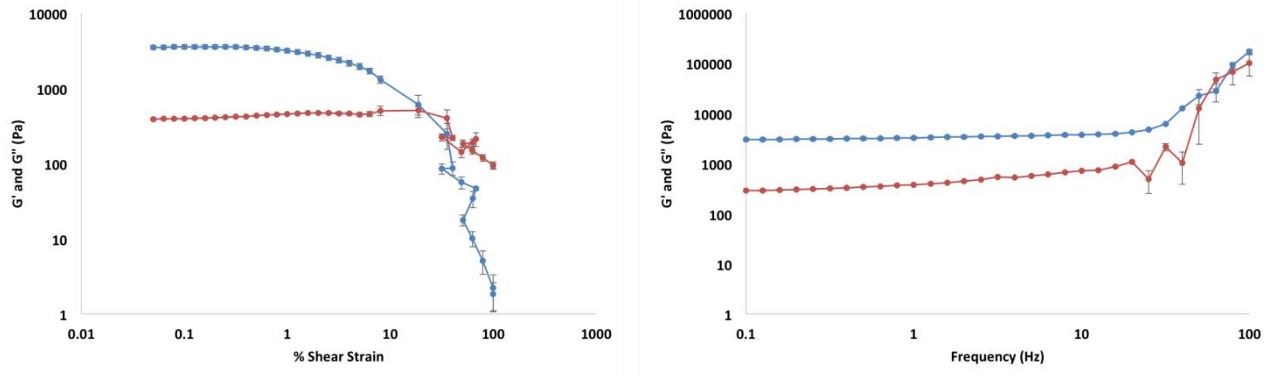

Figure S15. Elastic (G’, blue circles) and viscous (G”, red circles) moduli of DBS-CONHNH$_2$/alginate hydrogel (0.3% wt/vol DBS-CONHNH$_2$ and 0.1% wt/vol alginate) prepared with 0.15% wt/vol $CaCO_3$ and 0.8% wt/vol GdL with increasing shear strain (left) and frequency (right).

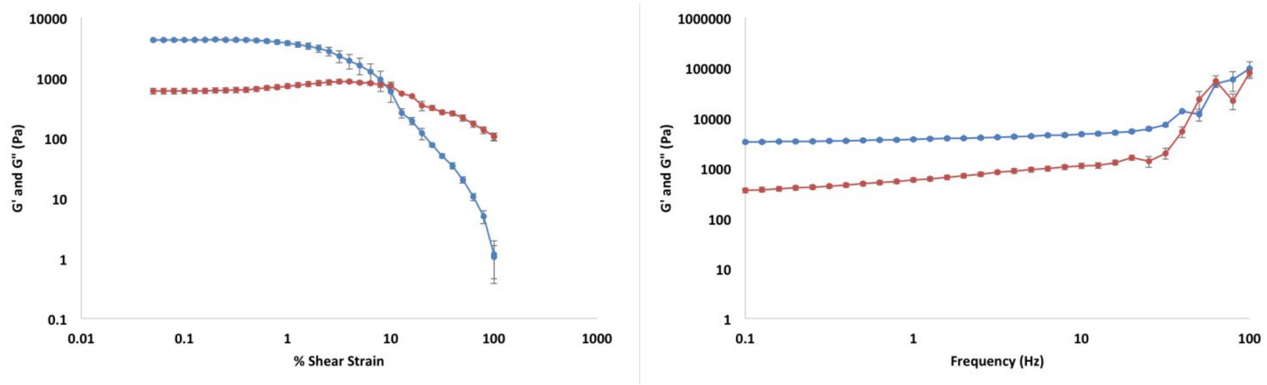

Figure S16. Elastic (G’, blue circles) and viscous (G”, red circles) moduli of DBS-CONHNH$_2$/alginate hydrogel (0.3% wt/vol DBS-CONHNH$_2$ and 0.3% wt/vol alginate) prepared with 0.15% wt/vol $CaCO_3$ and 0.8% wt/vol GdL with increasing shear strain (left) and frequency (right).

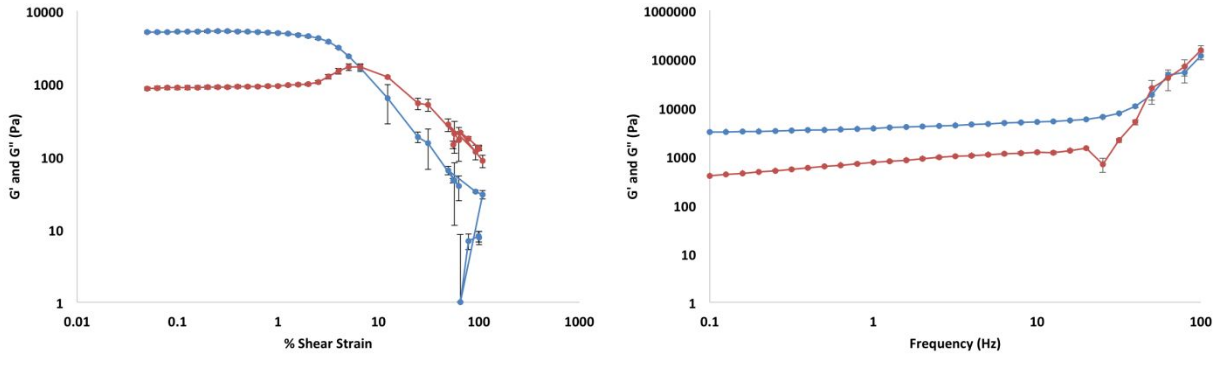

Figure S17. Elastic (G’, blue circles) and viscous (G”, red circles) moduli of DBS-CONHNH$_2$/alginate hydrogel (0.3% wt/vol DBS-CONHNH$_2$ and 0.5% wt/vol alginate) prepared with 0.15% wt/vol $CaCO_3$ and 0.8% wt/vol GdL with increasing shear strain (left) and frequency (right).

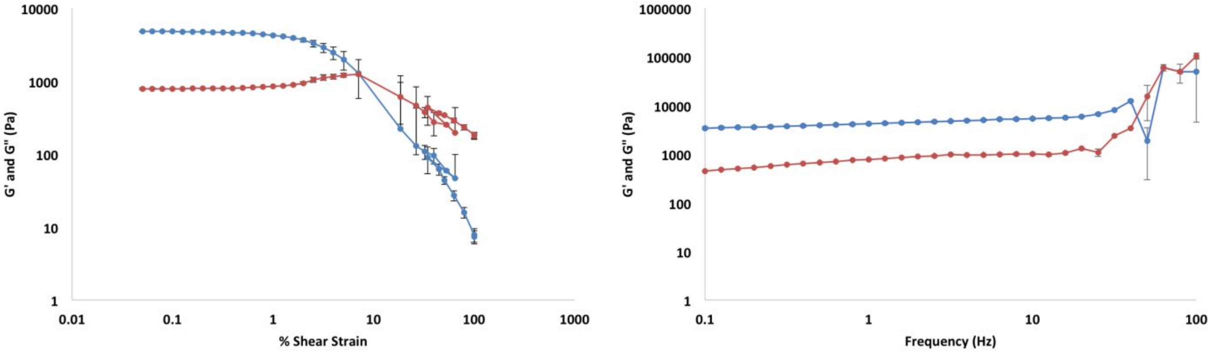

Figure S18. Elastic (G’, blue circles) and viscous (G”, red circles) moduli of DBS-CONHNH$_2$/alginate hydrogel (0.3% wt/vol DBS-CONHNH$_2$ and 1.0% wt/vol alginate) prepared with 0.15% wt/vol $CaCO_3$ and 0.8% wt/vol GdL with increasing shear strain (left) and frequency (right).

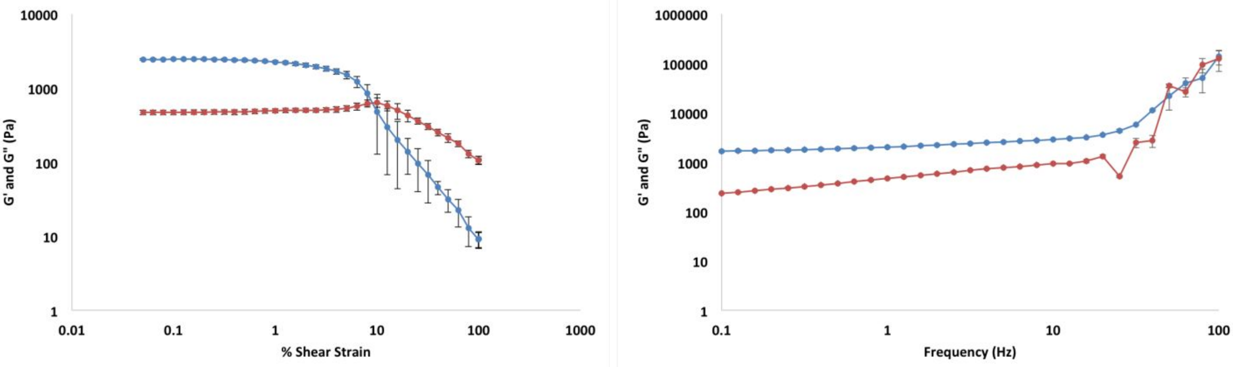

Figure S19. Elastic (G’, blue circles) and viscous (G”, red circles) moduli of DBS-CONHNH$_2$/alginate hydrogel (0.3% wt/vol DBS-CONHNH$_2$ and 0.3% wt/vol alginate) prepared with 0.05% wt/vol $CaCO_3$ and 0.8% wt/vol GdL with increasing shear strain (left) and frequency (right).

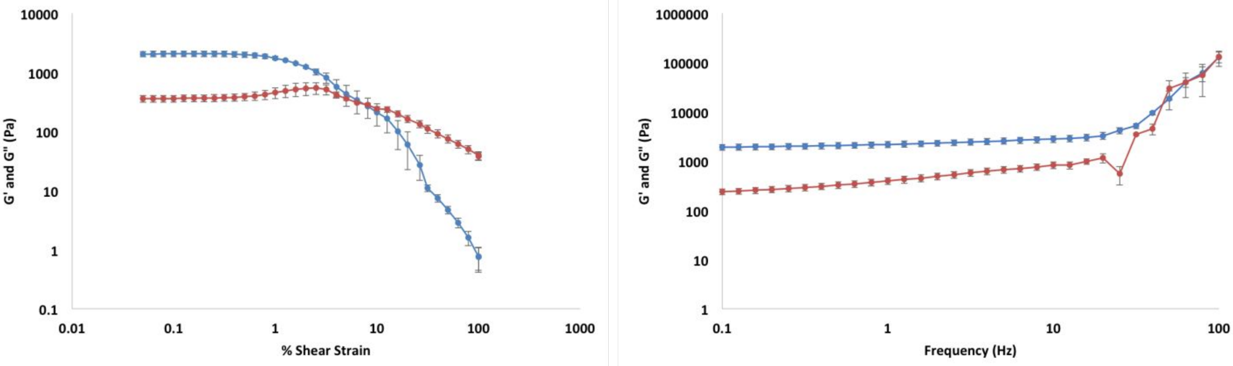

Figure S20. Elastic (G’, blue circles) and viscous (G”, red circles) moduli of DBS-CONHNH$_2$/alginate hydrogel (0.3% wt/vol DBS-CONHNH$_2$ and 0.3% wt/vol alginate) prepared with 0.3% wt/vol $CaCO_3$ and 0.8% wt/vol GdL with increasing shear strain (left) and frequency (right).

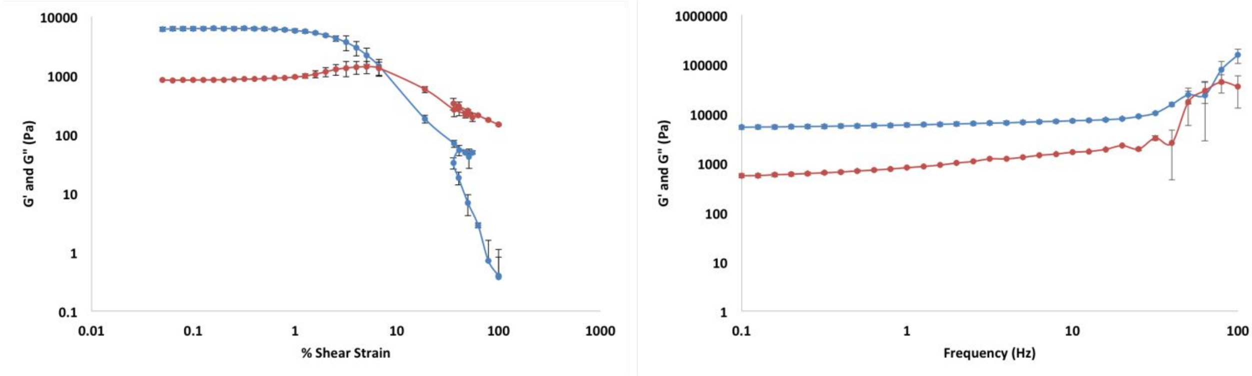

Figure S21. Elastic (G', blue circles) and viscous (G", red circles) moduli of DBS-CONHNH$_2$/alginate hydrogel (0.3% wt/vol DBS-CONHNH$_2$ and 0.3% wt/vol alginate) prepared with 0.15% wt/vol $CaCO_3$ and 1.0% wt/vol GdL with increasing shear strain (left) and frequency (right).

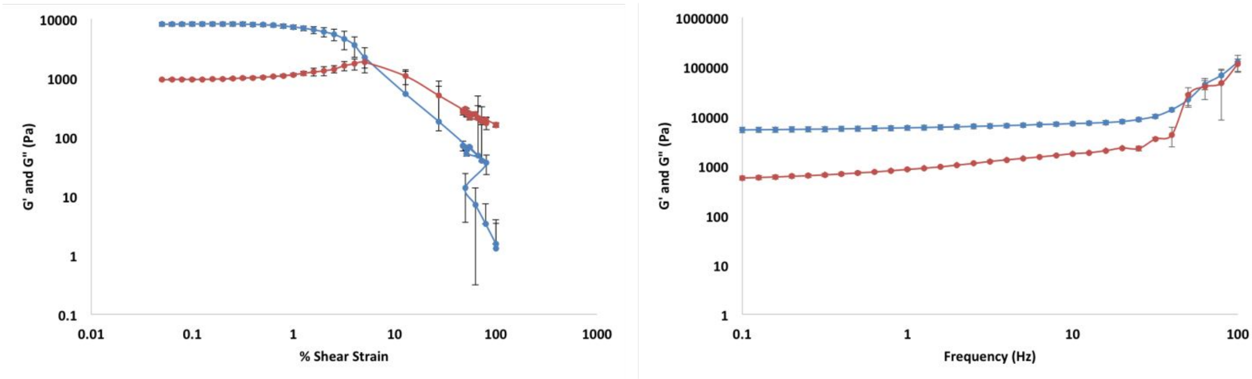

Figure S22. Elastic (G', blue circles) and viscous (G", red circles) moduli of DBS-CONHNH$_2$/alginate hydrogel (0.3% wt/vol DBS-CONHNH$_2$ and 0.3% wt/vol alginate) prepared with 0.15% wt/vol $CaCO_3$ and 1.2% wt/vol GdL with increasing shear strain (left) and frequency (right).

S3 Preparation and characterisation of UV responsive DBS-CONHNH$_2$/alginate and alginate gels

S3.1 Gel preparation and photopatterning

S3.1.1 Preparation of DBS-CONHNH$_2$/alginate UV responsive gels using DPIN as a pH activator. DBS-CONHNH$_2$ (0.3% wt/vol in 1 mL final total volume) and $CaCO_3$ (0.15% wt/vol in 1 mL final total volume) were suspended in water (0.5 mL). The suspension was sonicated to help the dispersion of the solid particles and subsequently mixed with a DPIN aqueous solution (0.8% wt/vol in 1 mL final total volume), which was acidified by addition of a 1 M HCl solution (2.5 μL). An aqueous alginate solution (1.0% wt/vol - 0.5 mL) was then added. The amount of alginate and water was adjusted depending on the desired final concentration of the polymer in the different experiments. The resulting suspension was heated until complete dissolution of the DBS-CONHNH$_2$ and then placed in ice and exposed to UV light for 2 hours to allow gel formation.

S3.1.2 Preparation of UV responsive alginate gels using diphenyliodonium nitrate (DPIN) as a pH activator. Alginate gels were prepared by adding $CaCO_3$ (0.15% wt/vol in 1 mL final total volume) to an aqueous alginate solution (0.4-1.3% wt/vol). The suspension was sonicated to help the dispersion of the $CaCO_3$ solid particles and subsequently mixed with a DPIN aqueous solution (0.8% wt/vol in 1 mL final total volume),

which was acidified by addition of a 1 M HCl solution (2.5 μL). The amount of alginate and water was adjusted depending on the desired final concentration of the polymer in the different experiments. The sample was subsequently placed in ice and exposed to UV light for 2 hours to allow gel formation.

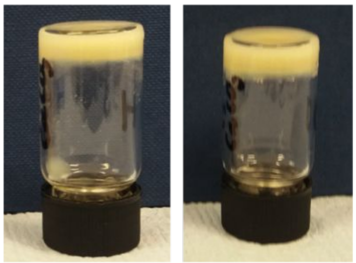

Figure S23. Photographic images of DBS-CONHNH$_2$/alginate (left) and alginate gels (right) prepared by photo-activation using $CaCO_3$ (0.15% wt/vol) and DPIN (0.8% wt/vol).

S3.1.3 Preparation of photopatterned DBS-CONHNH$_2$/alginate gels in trays. DBS-CONHNH$_2$ (0.3% wt/vol in 5 mL final total volume) and $CaCO_3$ (0.15% wt/vol in 5 mL final total volume) were suspended in 3.5 mL of a DPIN aqueous solution (0.8% wt/vol in 5 mL final total volume), which was acidified by addition of a 1 M HCl solution (12.5 μL). The suspension was sonicated to help the dispersion of the solid particles and subsequently mixed with an aqueous alginate solution (1.0% wt/vol - 1.5 mL). The resulting suspension was heated until complete dissolution of the DBS-CONHNH$_2$. The hot solution was then quickly transferred into a 5 x 5 cm glass tray. The sample was left undisturbed for 15 minutes to allow the formation of the DBS-CONHNH$_2$ network. A laser printed mask was then placed on top of the glass tray and the gel was exposed to UV light for two hours. To avoid the disruption of gelation due to heating effects, ice was placed below the glass tray.

S3.2 NMR assay

S3.2.1 *DBS-CONHNH$_2$/alginate two-component gels prepared using DPIN as a pH activator.* ^{1}H NMR was employed to validate the efficacy of the gel preparation method and to confirm that the two gelators were in self-assembled state. The gels used in these experiment were prepared by combining DBS-CONHNH$_2$ (0.3 % wt/vol) and alginate (0.5 % wt/vol) in D_2O (0.7 mL) as described in Section S3.1.1. DMSO (1.4 μL) was added as an internal standard. The gels were formed directly in NMR tubes and analysed by ^{1}H NMR after being exposed to the UV light for 2 hours.

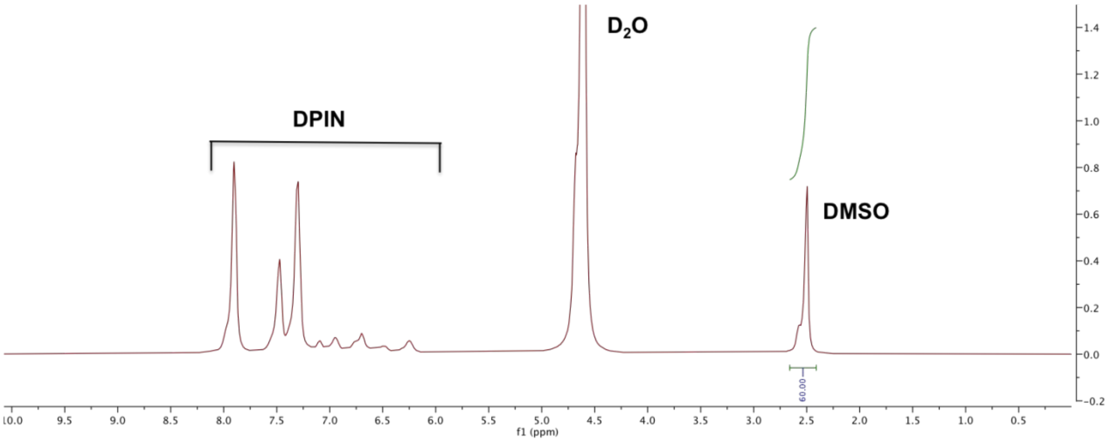

Figure S24. ^{1}H NMR of DBS-CONHNH$_2$/alginate gel prepared using $CaCO_3$ as a cross-linker and DPIN as a pH activator.

S3.3 Transmission and Scanning Electron Microscopy (TEM and SEM)

Samples for TEM and SEM imaging were prepared in sample vials as described in Section S3.1 and treated for analysis as described in Section S2.6.

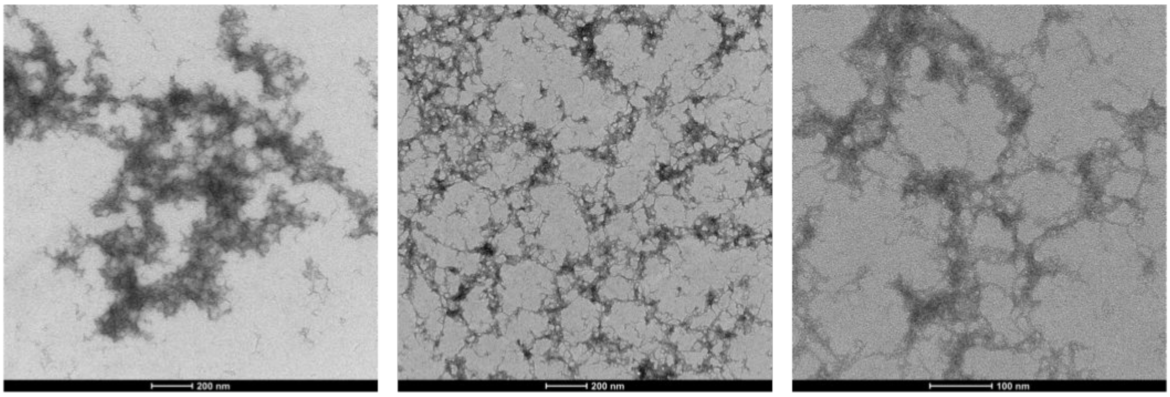

Figure S25. TEM image of photoactivated DBS-CONHNH$_2$/alginate hybrid gel (left) and alginate gel (centre and right) prepared using $CaCO_3$ as a cross-linker and DPIN as a pH activator. Scale bars: 200 nm (left and centre) and 100 nm (right).

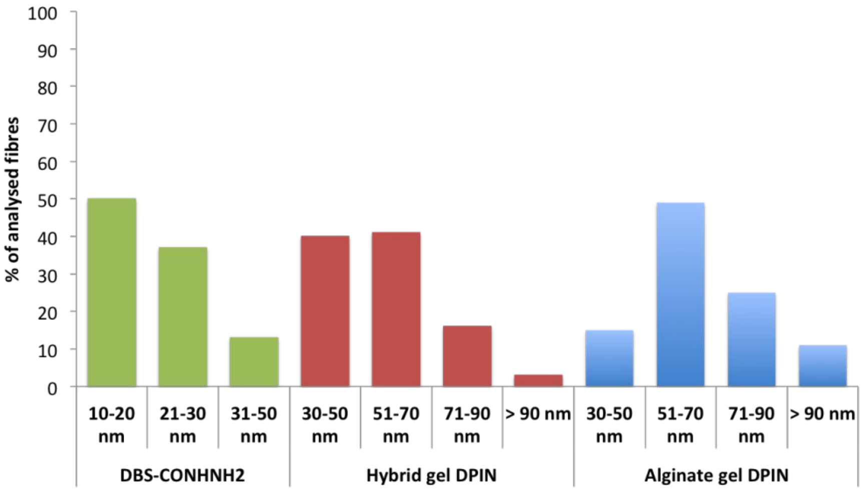

Figure S26. Fibre widths of DBS-CONHNH$_2$, DBS-CONHNH$_2$/alginate hybrid and alginate photoactivated gels prepared using $CaCO_3$ as a cross-linker and DPIN as a pH activator.

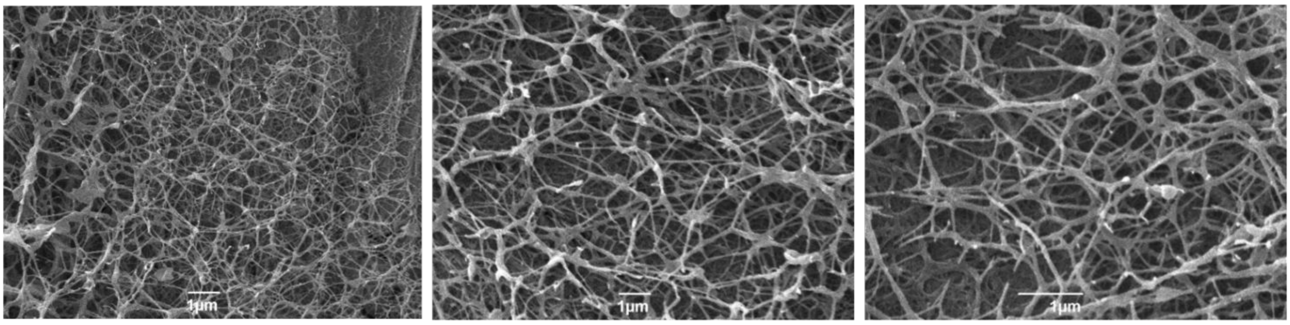

Figure S27. SEM images of photoactivated DBS-CONHNH$_2$/alginate hybrid (left) and alginate gel (centre and right) prepared using $CaCO_3$ as a cross-linker and DPIN as a pH activator. Scale bars: 1 μm.

S3.4 Thermal stability studies

All the gels for T_{gel} determination were prepared as described in Section S3.1 and T_{gel} values were obtained as descried in Section 2.7. These experiments were performed in triplicate to ensure reproducibility and the average is reported. Errors are estimated at ±2°C.

Table S3. T_{gel} values of photoactivated DBS-CONHNH$_2$/alginate and alginate gels prepared using $CaCO_3$ as a cross-linker (0.15% wt/vol) and DPIN (0.8% wt/vol) as a pH activator.

Gel (1 mL total volume)	Loading of DBS-CONHNH$_2$ (wt/vol)	Loading of ALGINATE (wt/vol)	T_{gel}
Alginate	-	0.8%	95 °C
DBS-CONHNH$_2$/alginate hybrid gel	0.3%	0.5%	>100 °C

S3.5 Rheology

Gel samples for rheology were prepared as described in Section S3.1 using bottomless vials and the experiments were carried out as described in Section S2.8.

Table S4. Rheological data as determined using oscillatory rheometry with a parallel plate geometry, for photoactivated DBS-CONHNH$_2$/alginate gels and calcium alginate gels. Loadings are given in wt/vol, and the G’/G” crossover points refer to the % shear strain at which G”=G’.

Gel	Loading of LMWG	Loading of Alginate	Total Loading	Loading of $CaCO_3$	Loading of DPIN	G’ (Pa)	G’/G” Crossover
Hybrid	0.3%	0.3%	0.6%	0.15%	0.8%	117	25.1%
Alginate	-	0.6%	0.6%	0.15%	0.8%	32.5	79.3%

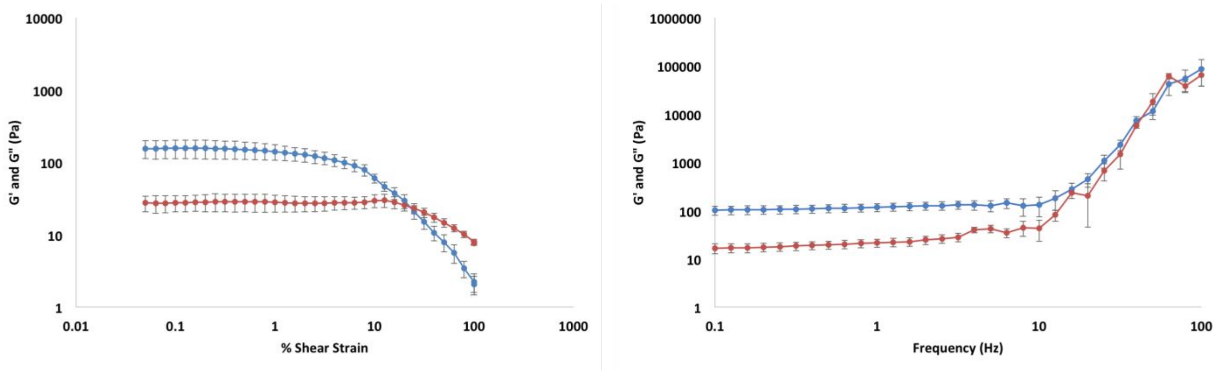

Figure S28. Elastic (G’, blue circles) and viscous (G”, red circles) moduli of photoactivated DBS-CONHNH$_2$/alginate hydrogel (0.3% wt/vol DBS-CONHNH$_2$ and 0.3% wt/vol alginate) prepared with 0.15% wt/vol $CaCO_3$ and 0.8% wt/vol DPIN with increasing shear strain (left) and frequency (right).

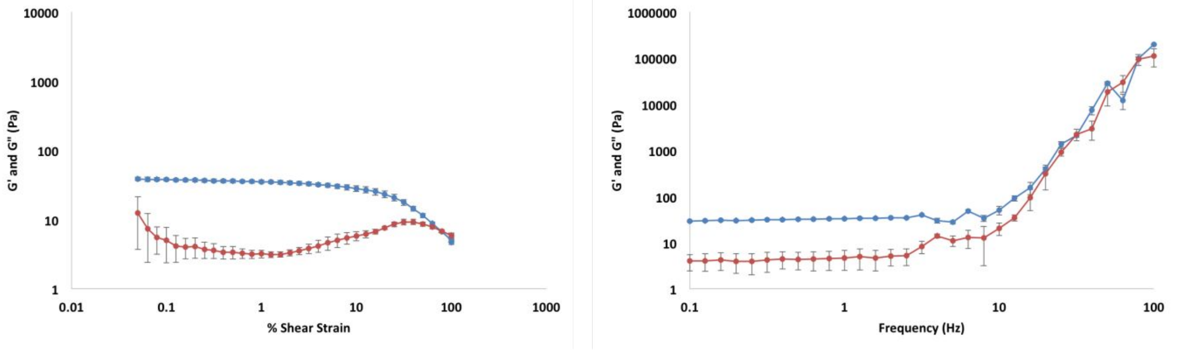

Figure S29. Elastic (G', blue circles) and viscous (G", red circles) moduli of photoactivated alginate hydrogel (0.6% wt/vol) prepared with 0.15% wt/vol $CaCO_3$ and 0.8% wt/vol DPIN with increasing shear strain (left) and frequency (right).

S4 Preparation and characterisation of DBS-$CONHNH_2$, DBS-$CONHNH_2$/alginate and alginate gels loaded with silver nanoparticles (AgNPs)

S4.1 *In situ* formation of AgNPs

To induce the *in situ* formation of AgNPs, each gel was thoroughly washed with water multiple times and immersed in 1 or 3 mL of $AgNO_3$ solution (10 mM) for 3 days. After 3 days, the supernatant was gently removed with a pipette and the gels were washed with water multiple times. A colour change was observed in the samples in which the Ag was reduced from Ag(I) to Ag(0).

S4.2 Uptake of Ag(I)

S4.2.1 *Uptake of Ag(I) into DBS-$CONHNH_2$ and alginate gels (prepared in sample vials).* The gels used to estimate the uptake of Ag(I) were prepared in water (1 mL) as described in section S2. Each of these gels was thoroughly washed with water multiple times and immersed in 1 or 3 mL of a 10 mM $AgNO_3$ solution (containing respectively 0.01 or 0.03 mmoles of Ag(I)) for 3 days. After 3 days, the supernatant was transferred into a vial and used to titrate 0.0005 mmol of NaCl (1 mL) in the presence of K_2CrO_4 (5% - 1 mL) as an indicator. To evaluate precisely the volume of titrant used, the titration was performed by slowly adding the titrant to the NaCl solution in 10 µL drops under stirring. The volume of supernatant added as a titrant, was used to calculate the mmoles of residual Ag(I) in the supernatant (*i.e.* the Ag(I) that was not incorporated into the gel). This was subtracted from the initial mmoles of Ag(I) added, to give the mmoles of Ag(I) incorporated into the gel. To ensure data reproducibility, this experiment was performed in triplicate for each gel and average values are reported.

S4.2.2 *Uptake of Ag(I) into DBS-$CONHNH_2$/alginate gel beads.* The gel beads used to estimate the uptake of Ag(I) were prepared as described in Section S2. Each gel bead was prepared in a 20 µL volume. Since ten gel beads per type of gel were prepared, a 200 µL total volume of gel (*i.e.* 20% of the volume used for the gels described in Section S4.1) was considered for the subsequent addition of $AgNO_3$ in the same proportion used for the other gels. Once ready, the gel beads were thoroughly washed with water multiple times and immersed in 0.2 or 0.6 mL of a 10 mM $AgNO_3$ solution (containing respectively 0.002 or 0.006 mmoles of Ag(I)) for 3 days. After 3 days, the supernatant was transferred into a vial and used to titrate a 0.0005 mmol of NaCl (1 mL) in the presence of K_2CrO_4 (5% - 1 mL) as an indicator. To evaluate precisely the volume of titrant used, the titration was performed by slowly adding the titrant to the NaCl solution in 10 µL drops under stirring. The volume of supernatant added as a titrant, was used to calculate the mmoles of residual Ag(I) in the supernatant (*i.e.* the Ag(I) that was not incorporated into the gel). This was subtracted to the

initial mmoles of Ag(I) added, to give the mmoles of Ag(I) incorporated into the gel. To ensure data reproducibility, this experiment was performed in triplicate for each gel and average values are reported.

Table S5. Evaluation of Ag (I) uptake into DBS-CONHNH$_2$, alginate gels and DBS-CONHNH$_2$/alginate hybrid gel beads by precipitation titration.

Gel	Loading of DBS-CONHNH$_2$ (wt/vol)	Loading of Alginate (wt/vol)	mmol AgNO$_3$ loaded onto gel	mmol Ag (I) incorporated into gel	mmol Ag (I) incorporated / mL of gel	% of Ag (I) incorporated
DBS-CONHNH$_2$	0.3 %	-	0.03	0.0167	0.0167	55.5 %
DBS-CONHNH$_2$	0.3 %	-	0.01	0.0068	0.0068	68.5 %
Alginate	-	0.8 %	0.03	0.011	0.011	36.7 %
Alginate	-	0.8 %	0.01	0.0048	0.0048	48.2 %
Hybrid gel (10B)	0.3 %	0.5 %	0.006	0.00298	0.015	50.0 %
Hybrid gel (10B)	0.3 %	0.5 %	0.002	0.00103	0.0065	65.0 %

S4.3 Release of Ag(I) from DBS-CONHNH$_2$/alginate gel beads loaded with Ag NPs

S4.3.1 Preparation of gel beads. The DBS-CONHNH$_2$/alginate gel beads (40 beads/sample) were prepared as described in section S2 using 20 µL volume/gel bead and washed with water multiple times. The *in situ* formation of Ag NPs was induced by immersing the gels in 3 ml of a 10 mM solution of AgNO$_3$ for 24 hours. After 24 hours, the supernatant was removed and used to calculate the exact amount of Ag (I) incorporated by precipitation titration as described in Section S4.2.2. To ensure reproducibility, three samples of 40 beads each were prepared for each time point and the exact amount of Ag (I) incorporated in each sample was calculated and reported in Table S5.

Table S6. Amount of Ag(I) incorporated in each sample of 40 DBS-CONHNH$_2$/alginate gel beads used for the release study.

Sample name	mmol Ag (I) incorporated into 40 gel beads	% of Ag (I) incorporated
A	0.013	44.4
B	0.016	54.5
C	0.015	50.0
D	0.011	37.5
E	0.013	44.4
F	0.015	50.0
G	0.015	50.0
H	0.016	54.5
I	0.015	50.0
J	0.013	44.4
K	0.013	44.4
L	0.011	37.5
M	0.011	37.5
N	0.016	54.5
O	0.016	54.5
P	0.015	50.0

Q	0.011	37.5
R	0.013	44.4
S	0.015	50.0
T	0.015	50.0
U	0.016	54.5
V	0.013	44.4
W	0.011	37.5
X	0.023	77.3

S4.3.2 Release of Ag(I) from DBS-CONHNH$_2$/alginate gel beads. Each sample (40 gel beads/sample) was immersed in 2 ml of water. At the specified time intervals, the release medium was removed and used to titrate 0.0005 mmol of NaCl (1 mL) in the presence of K_2CrO_4 (5% - 1 mL) as an indicator. To evaluate precisely the volume of titrant used, the titration was performed by slowly adding the titrant to the NaCl solution in 10 µL drops under stirring. The volume of supernatant added as a titrant, was used to calculate the mmoles of released Ag (I) (Table S6). To ensure data reproducibility, this experiment was performed in triplicate for each gel and average values were reported in the final graph.

Table S7. Ag(I) released from each sample.

Sample name	Time point (hours)	Mmoles of Ag (I) released from gel beads	% of Ag (I) released	Average %	Standard error
A	0.5	0.0026	19.7	17.7	2.3
B	0.5	0.0025	15.3		
C	0.5	0.0028	18.5		
D	1	0.0025	22.2	18.8	2.85
E	1	0.0024	18.3		
F	1	0.0025	16.7		
G	1.5	0.0024	15.9	16.3	1.02
H	1.5	0.0026	15.7		
I	1.5	0.0026	17.5		
J	2	0.0024	18.3	19.1	2.09
K	2	0.0024	17.8		
L	2	0.0024	21.7		
M	4	0.0026	23.4	17.4	4.77
N	4	0.0027	16.5		
O	4	0.0023	14.2		
P	6	0.0024	15.9	18.2	2.95
Q	6	0.0024	21.7		
R	6	0.0024	17.9		
S	8	0.0024	15.9	16.2	1.17
T	8	0.0026	17.5		
U	8	0.0025	15.3		
V	24	0.0024	17.9	15.8	5.73
W	24	0.0026	22.8		
X	24	0.0026	11.3		

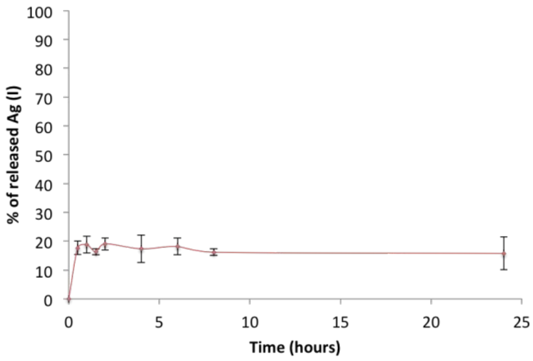

Figure S30. Release over time of Ag(I) ions from the DBS-CONHNH$_2$/alginate hybrid gel beads.

S4.4 Transmission Electron Microscopy (TEM)

Samples for TEM imaging were prepared as described in Section S2, loaded with Ag NPs (Section S4.1) and then treated for TEM analysis as described in Section S2.6.

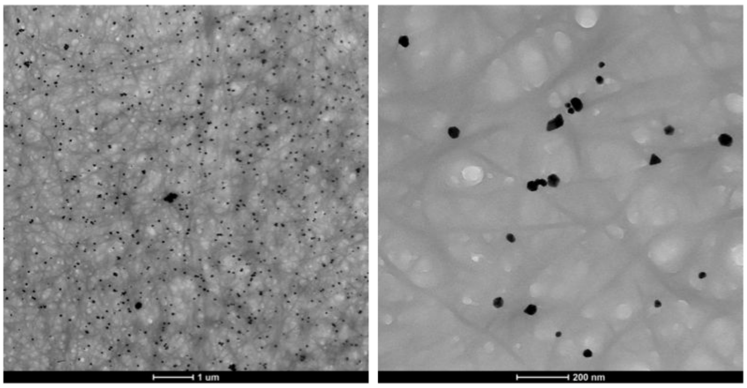

Figure S31. TEM images of DBS-CONHNH$_2$ gel incorporating Ag NPs. Scale bars: 1 µm and 200 nm.

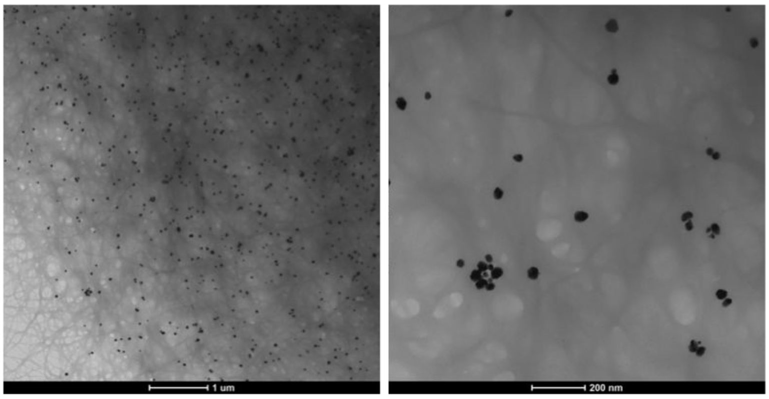

Figure S32. TEM images of DBS-CONHNH$_2$/alginate gel beads incorporating Ag NPs. Scale bars: 1 µm and 200 nm.

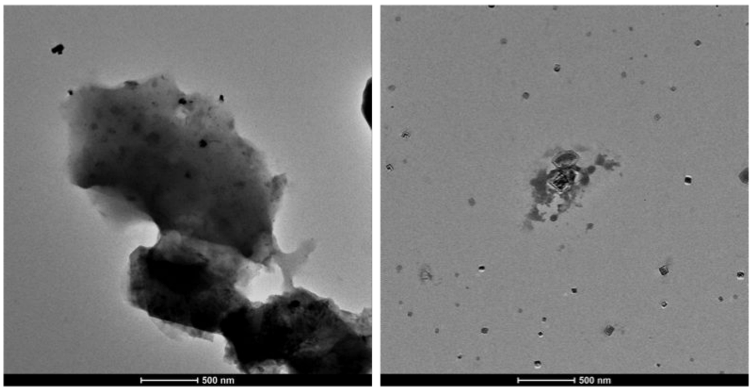

Figure S33. TEM images of alginate gel incorporating Ag NPs. Scale bars: 500 nm.

S4.5 Rheology

Gel samples for rheology were prepared in bottomless vials, using a 0.15% wt/vol $CaCO_3$ concentration and a 0.8% wt/vol GdL concentration, as described in Section S2. Once ready, the gels were washed with water and then treated with 1 or 3 mL of a 10 mM $AgNO_3$ solution (containing respectively 0.01 or 0.03 mmoles of Ag (I)) for 3 days. After 3 days, the supernatant was removed and the mechanical properties of the gels were analysed as described in Section S2.8.

Table S8. Rheological data as determined using oscillatory rheometry with a parallel plate geometry, for DBS-CONHNH$_2$, DBS-CONHNH$_2$/alginate gels and calcium alginate gels with Ag NPs. Loadings are given in wt/vol, and the G'/G" crossover points refer to the % shear strain at which G"= G'.

Gel	Loading of LMWG	Loading of Alginate	Total Loading	Volume of $AgNO_3$ (10 mM) added	G' (Pa)	G'/G" Crossover
DBS-CONHNH$_2$	0.4%	-	0.4%	1 mL	9.72	39.7%
DBS-CONHNH$_2$	0.4%	-	0.4%	3 mL	7.79	31.5%
Hybrid	0.3%	0.5%	0.8%	1 mL	1320	3.1%
Hybrid	0.3%	0.5%	0.8%	3 mL	584	6.3%
Alginate	-	0.8%	0.8%	1 mL	385	12.6%
Alginate	-	0.8%	0.8%	3 mL	524	7.9%

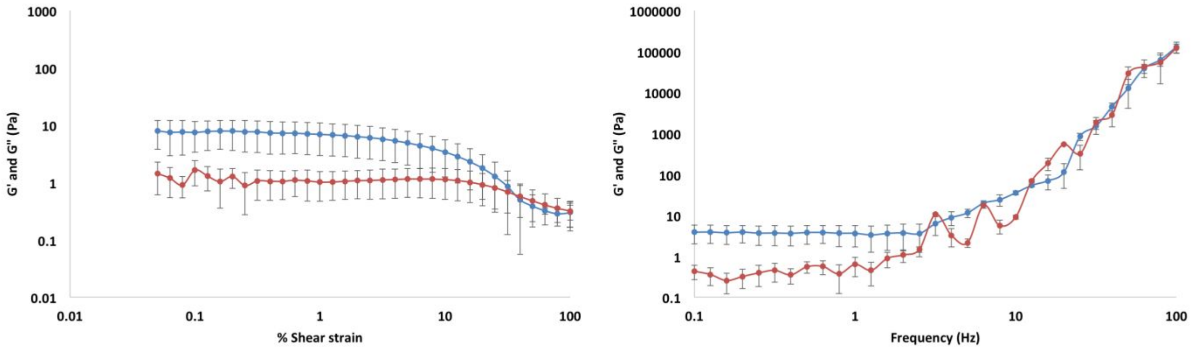

Figure S34. Elastic (G', blue circles) and viscous (G", red circles) moduli of DBS-CONHNH$_2$ hydrogel (0.4% wt/vol - loaded with 1 mL $AgNO_3$ 10 mM) with increasing shear strain (left) and frequency (right).

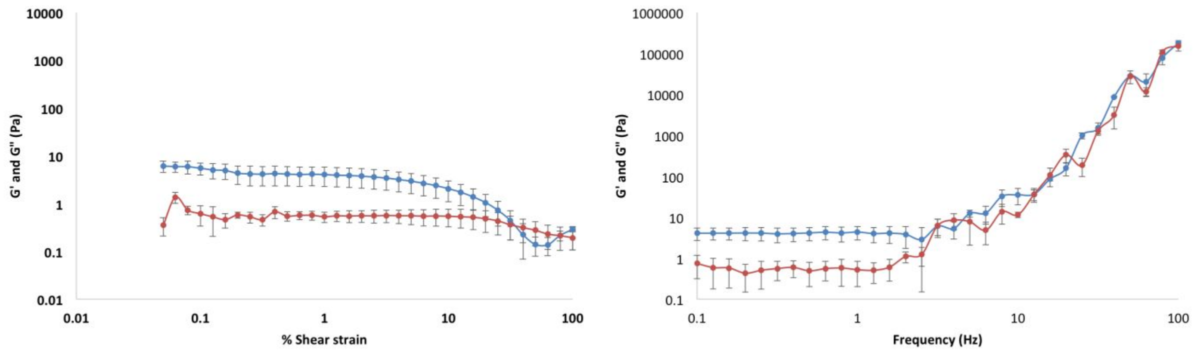

Figure S35. Elastic (G', blue circles) and viscous (G", red circles) moduli of DBS-CONHNH$_2$ hydrogel (0.4% wt/vol - loaded with 3 mL AgNO$_3$ 10 mM) with increasing shear strain (left) and frequency (right).

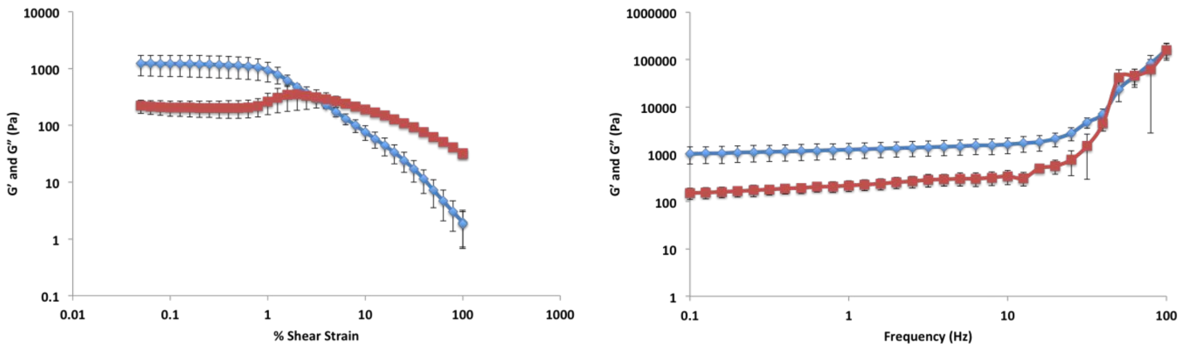

Figure S36. Elastic (G', blue circles) and viscous (G", red circles) moduli of DBS-CONHNH$_2$/alginate hydrogel (0.3% wt/vol DBS-CONHNH$_2$ and 0.5% wt/vol alginate – loaded with 1 mL AgNO$_3$ 10 mM) with increasing shear strain (left) and frequency (right).

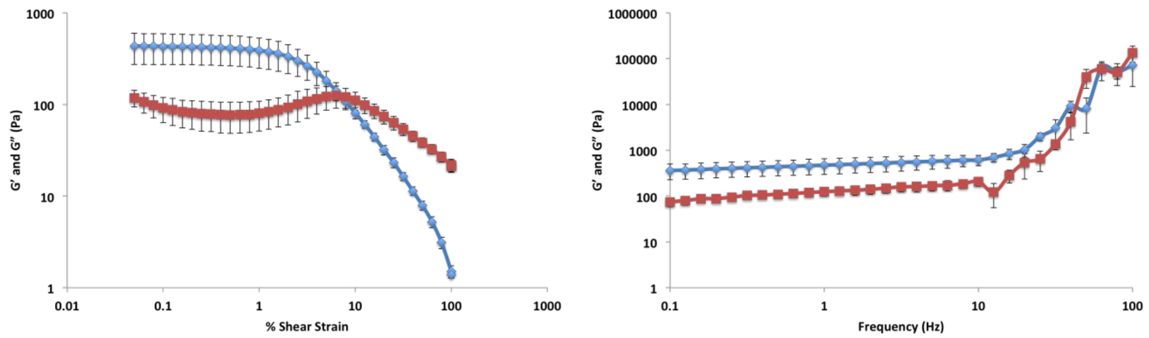

Figure S37. Elastic (G', blue circles) and viscous (G", red circles) moduli of DBS-CONHNH$_2$/alginate hydrogel (0.3% wt/vol DBS-CONHNH$_2$ and 0.5% wt/vol alginate – loaded with 3 mL AgNO$_3$ 10 mM) with increasing shear strain (left) and frequency (right).

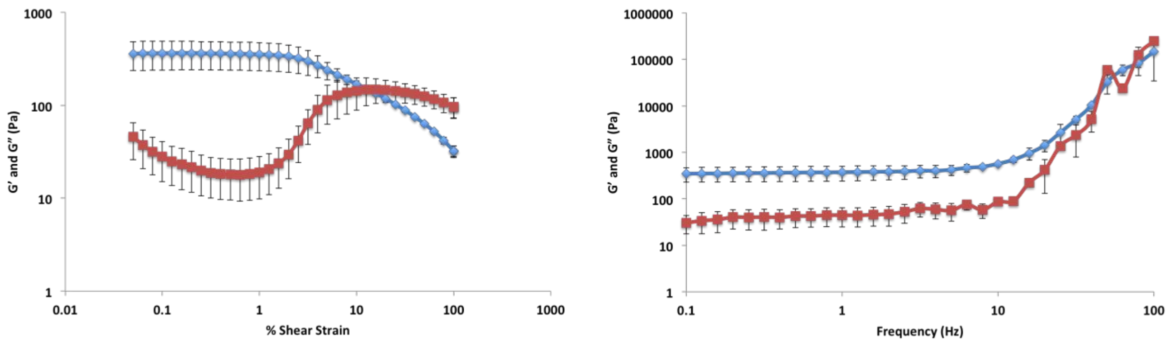

Figure S38. Elastic (G', blue circles) and viscous (G", red circles) moduli of alginate hydrogel (0.8% wt/vol– loaded with 1 mL $AgNO_3$ 10 mM) with increasing shear strain (left) and frequency (right).

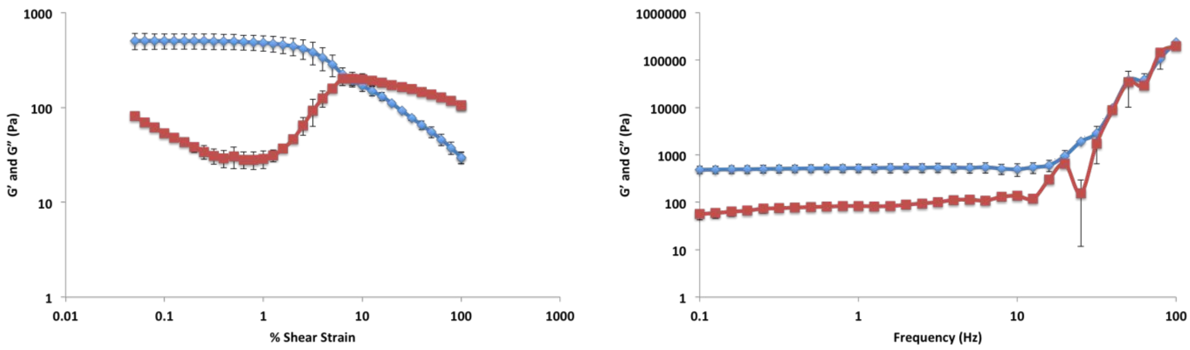

Figure S39. Elastic (G', blue circles) and viscous (G", red circles) moduli of alginate hydrogel (0.8% wt/vol– loaded with 3 mL $AgNO_3$ 10 mM) with increasing shear strain (left) and frequency (right).

S5 Biological studies

S5.1 Cell line

Y201 immortalised human mesenchymal stem cells (MSCs)[3] were grown in a T175 flask with Dulbecco's Modified Eagle's Medium (DMEM) with fetal bovine serum (FBS - 10%) and penicillin/streptomycin (P/S - 1%). To obtain the cells, the medium was removed from the flask and the cells washed with Dulbecco's phosphate buffer saline solution (11 mL). Trypsin/EDTA (2 mL) was then added and the cells were incubated at 37°C for approximately five mins. When cell detachment was observed by optical microscopy, trypsin was neutralised with 9 mL DMEM (10% FBS, 1% P/S). The cells were then transferred in a tube and isolated by centrifugation. After centrifugation, the supernatant was removed and the cell pellet was dispersed in 5 mL DMEM (10% FBS, 1% P/S). Cell count was performed using a Countess Automated Cell Counter (Thermo Fisher) on a 10 μL aliquot of a stock solution obtained by mixing 20 μL of cell suspension with 20 μL of trypan blue.

S5.2 Cytotoxicity assay

S5.2.1 Gel preparation. DBS-CONHNH$_2$/alginate hybrid gels (0.3% wt/vol DBS-CONHNH$_2$ and 0.5% wt/vol alginate) and alginate gels (0.8% wt/vol) for cytotoxicity assays were prepared in triplicate in a 48-well plate (300 μL volume), in the presence of $CaCO_3$ (0.15% wt/vol) and GdL (1.0% wt/vol), as described in Section S2. The *in situ* formation of Ag NPs was induced by addition of an $AgNO_3$ solution (300 μL – 0.0125 mM or 10 mM) on top of the gels. The gels were left undisturbed for 72 hours, subsequently washed with Dulbecco's Modified Eagle's Medium (DMEM) multiple times (400 μL) and then transferred in the middle of a 6-well plate. DBS-CONHNH$_2$ gels were directly prepared in triplicate in a 6-well plate (300 μL volume;

0.3% wt/vol), using small bottomless vials (c.a. 1 cm diameter). Once the gels were formed, the vials were removed, leaving self-supporting gels in the middle of each well.

S5.2.2 Plate seeding. The cells (100000/well) were seeded on the bottom of the wells around the gels in the 6-well plates and covered with DMEM (10% FBS, 1% P/S - 2 μL).

S5.2.3 Crystal violet staining. After 48 hours, the DMEM was removed and each well was washed with PBS (1 mL). A crystal violet methanol solution (1 mL) was added to each well and the plates were left undisturbed for 20 mins. After 20 mins, the stain was collected and the plates were washed multiple times in a distilled water bath and then left to dry. Plates were imaged with an Epson PhotoScanner.

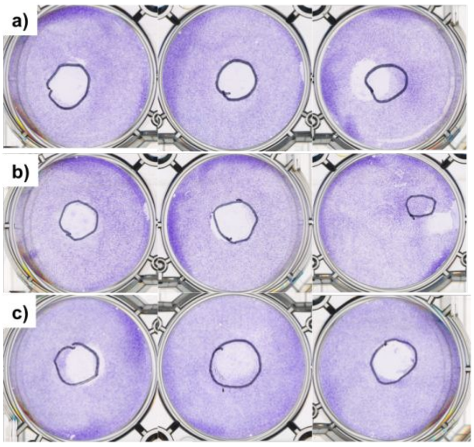

Figure S40. Scanned images of the cytotoxicity assay. Control gels without AgNPs. (a) DBS-CONHNH$_2$/alginate gel, (b) alginate gel, (c) DBS-CONHNH$_2$ gel.

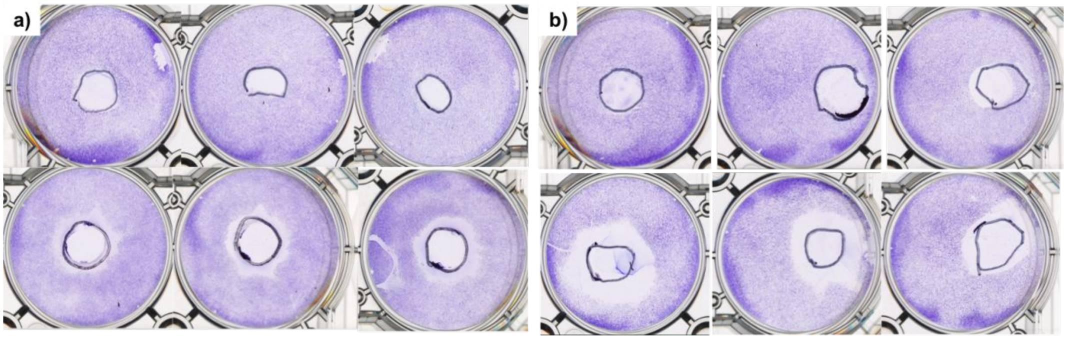

Figure S41. Scanned images of the cytotoxicity assay. Gels loaded with AgNPs (0.0125 mM top and 10 mM bottom), the white rings around the gels indicate the zone of cell growth inhibition. (a) DBS-CONHNH$_2$/alginate gel, (b) alginate gel.

S5.3 Viability assay

S5.3.1 Gel preparation in 96-well plates and plate seeding. Gels were prepared as described in Section S2 in 96-well plates in 75 μL volume. Since the gel beads were prepared using 20 μL volume per bead, four gel beads were placed in well. The *in situ* formation of Ag NPs was induced by addition of an $AgNO_3$ solution (75 μL – 0.00625, 0.0125, 0.05, 0.1, 1.0 or 10 mM) on top of the gels. The gels were left undisturbed for 72 hours. After this time, the supernatant was removed and the gels were washed multiple times with DMEM (10% FBS, 1% P/S - 200 μL). After the last wash, the gels were soaked with DMEM (10% FBS, 1% P/S - 100 μL) and the cells (25000/well) were seeded and covered with further DMEM (10% FBS, 1% P/S - 100 μL).

S5.3.2 Alamar Blue viability assay. Cell viability was measured at different time points using the Alamar Blue viability assay (*Thermo Fisher Scientific*). The cell culture medium was removed from each well and a 10% solution of Alamar Blue in DMEM (100 μL) was added. The plates were incubated at 37°C for 4 hours. After this time, 20 μL aliquots were taken from each well and diluted with DMEM (180 μL) in a new 96 well plate. Fluorescence was then measured with a fluorimeter (excitation 530-560 nm and emission 590 nm). This experiment was performed in sixuplicates and average values are reported with the error bars representing standard error. Control experiments with no cells were performed for each gel type.

S6 Disc diffusion assays

S6.1 Gel preparation

The gels were prepared in sterile conditions in 75 μL volume in a 96 well plate as described in Section S2. The *in situ* formation of Ag NPs was induced by immersing the gels in 75 μL of a 10 mM solution of $AgNO_3$ for 3 days. After 3 days, the supernatant was gently removed and the gels were carefully washed with water multiple times. The gels were then transferred on the agar plates, where the bacteria were cultured, for the disc diffusion assay.

S6.2 Disc diffusion assay

Cells from glycerol stocks were used to innoculate LB medium (5 mL; 10 g L^{-1} tryptone, 5 g L^{-1} yeast, 10 g L^{-1} NaCl), performed in triplicate for each strain. Cultures were grown at 37 °C with shaking at 180 rpm for 17 h. Cultures were diluted to OD600 0.1, followed by a further 1:100 dilution, with autoclaved water. These cultures were spread onto LB agar (75 μL per plate; 10 g L^{-1} tryptone, 5 g L^{-1} yeast, 10 g L^{-1} NaCl, 15 g L^{-1} agar). A 200 μL pipette tip was used to remove discs from the agar, into which gels were inserted. Plates were incubated at 37°C for 24 h before inhibition radii were measured. Bacterial Strains: vancomycin-resistant Enterococcus faecium (VRE): (https://www.dsmz.de/collection/catalogue/details/culture/dsm-17050), DSM 17050. Pseudomonas aeruginosa (PA14).

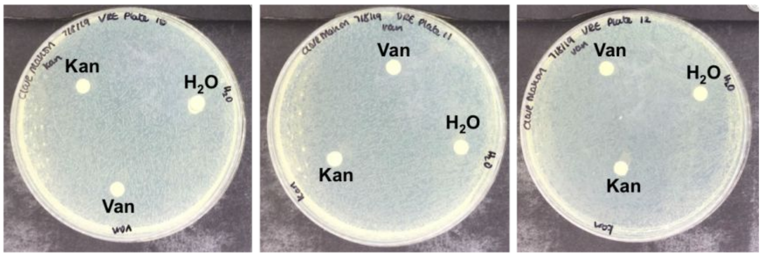

Figure S42. Disc diffusion assay - vancomycin-resistant E. faecium (VRE). Controls: water (H_2O), kanamycin (Kan), vancomycin (Van).

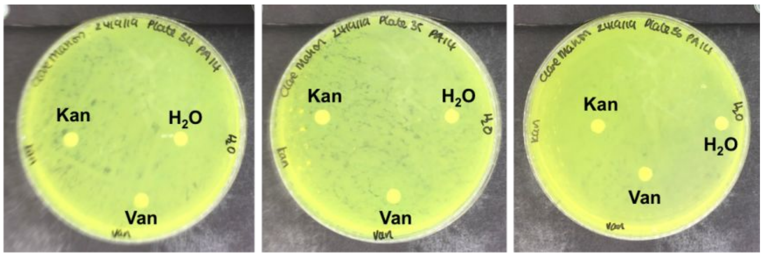

Figure S43. Disc diffusion assay – P. aeruginosa (PA14). Controls: water (H_2O), kanamycin (Kan), vancomycin (Van).

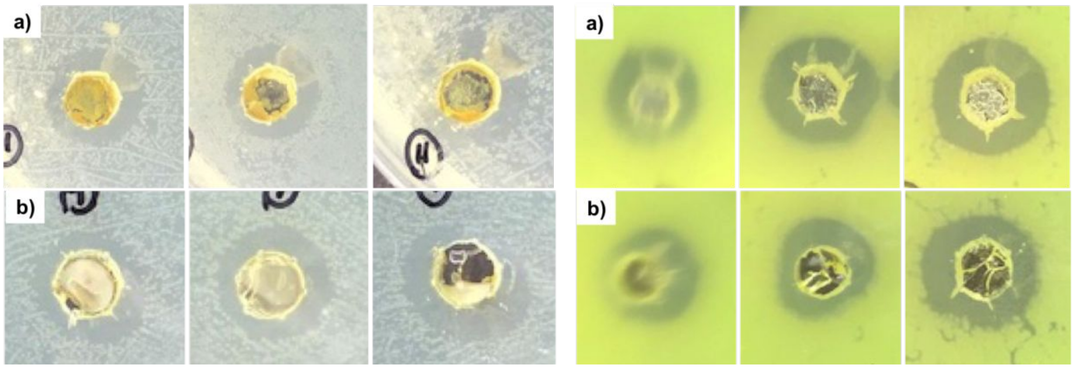

Figure S44. Photographic images of the disc diffusion assay. (Left) Vancomycin resistant *Enterococcus faecium* (VRE). (Right) *Pseudomonas aeruginosa*(PA14). Gels loaded with AgNPs, the dark rings indicate the zone of inhibition. (a) DBS-CONHNH$_2$ gel and (b) alginate gel. Images are 10 mm x 10 mm.

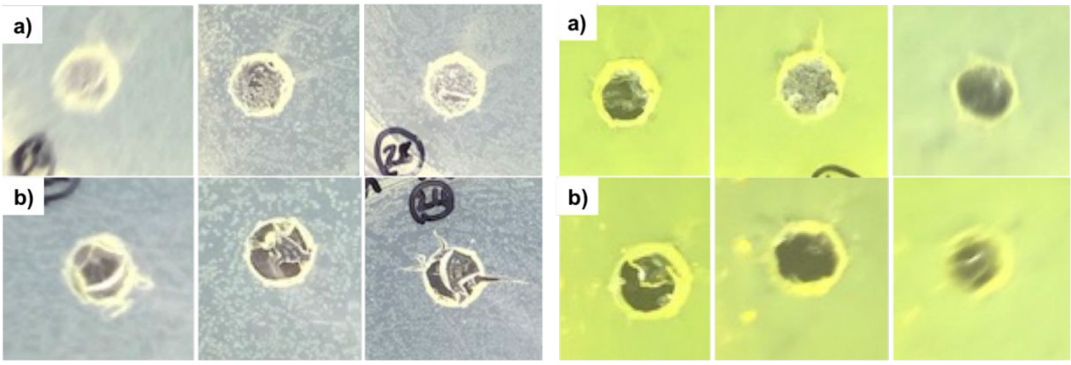

Figure S45. Photographic images of the disc diffusion assay. (Left) Vancomycin resistant *Enterococcus faecium* (VRE). (Right) *Pseudomonas aeruginosa*(PA14). Control gels without AgNPs. (a) DBS-CONHNH$_2$ gel, and (b) alginate gel. Images are 10 mm x 10 mm.

Table S9. Inhibition Radii from disc diffusion assay.

**Gel**	**Vancomycin-resistant Enterococcus (VRE)**		**Pseudomonas Aeruginosa (PA14)**	
	**Inhibition radii - Mean (mm)**	**Standard Error**	**Inhibition radii - Mean (mm)**	**Standard Error**
DBS-CONHNH$_2$	3.33	0.33	3.67	0.33
DBS-CONHNH$_2$– no Ag NPs	No inhibition	No inhibition	No inhibition	No inhibition
DBS-CONHNH$_2$/Alginate	3.33	0.33	3.33	0.33
DBS-CONHNH$_2$/Alginate – no Ag NPs	No inhibition	No inhibition	No inhibition	No inhibition
Alginate	3.00	0.00	3.00	0.00
Alginate – no Ag NPs	No inhibition	No inhibition	No inhibition	No inhibition

S7 References

[1] Okesola, B. O.; Smith, D. K. *Chem. Commun.*, **2013**, *49*, 11164-11166.
[2] Cornwell, D. J.; Okesola, B. O.; Smith, D. K. *Soft Matter*, **2013**, *9*, 8730-8736.
[3] James, S.; Fox, J.; Afsari, F.; Lee, J.; Clough, S.; Knight, C.; Ashmore, J.; Ashton, P.; Preham, O.; Hoogduijn, M.; Ponzoni, R. D. R.; Hancock, Y.; Coles, M.; Genever, P. *Stem Cell Reports*, **2015**, *4*, 1004-1015.
